# Gross anatomy of the skeleton of neonates of the Orinoco Matamata turtle (*Chelus orinocensis*)

**DOI:** 10.1371/journal.pone.0346436

**Published:** 2026-04-01

**Authors:** Liliana Andrea Valencia-Flórez, Luz Natalia Franco-Montoya, Lynda J. Tamayo-Arango, Claudia P. Ceballos

**Affiliations:** 1 Grupo de Investigación GAMMA, Escuela de Medicina Veterinaria, Facultad de Ciencias Agrarias, Universidad de Antioquia UdeA, Medellín, Colombia; 2 Grupo de Investigación Biogénesis, Escuela de Medicina Veterinaria, Facultad de Ciencias Agrarias, Universidad de Antioquia UdeA, Medellín, Colombia; 3 Grupo de Investigación CIBAV, Escuela de Medicina Veterinaria, Facultad de Ciencias Agrarias, Universidad de Antioquia UdeA, Medellín, Colombia; Chinese Academy of Sciences, CHINA

## Abstract

The Orinoco Matamata turtle (*Chelus orinocensis*) was described in 2020, representing the second evolutionary lineage within the genus *Chelus*. The anatomy of this species has not been described, and such information is essential for understanding its biology, ecology, and for its potential relevance in clinical diagnosis. This research provides the first description of the macroscopic morphology of the skeletal system in neonates of the Orinoco Matamata turtle. A group of confiscated turtles was donated from the Alexander von Humboldt institute to our university for academic purposes. From this group, we obtained the skeleton of ten individuals, which measured between 51–80 mm of straight carapace length. Then, the skeletons of the two largest individuals were disarticulated to describe each bone in detail based on anatomical atlases and turtle osteological guides. Bones from the cranium, mandible, hyoid apparatus, and the axial and appendicular skeletons were identified and photographed. Compared to other turtle species, some bones were absent, not fully ossified, or unusually well developed at this early life stage, likely reflecting ecological adaptations. Further ontogenetic studies on skeletal ossification are important to fully understand the evolutionary history and functional adaptations of this species.

## Introduction

The skeletal anatomy of turtles is well characterized in most cases as demonstrated by the descriptions available for *Apalone spinifera*, *Chelydra serpentina*, *Macrochelys temminckii*, *Araripemys barretoi* or *Phrynops hilarii* [1–5]. However, most of the studies have focused on specific regions of the body, for example, the cranium in *Chelus fimbriata* [6] and several species of Chelidae [7,8], the carapace of *Emydura subglobosa, Pelusios sinuatus, Pelomedusa subrufa* and *Podocnemis unifilis* [9,10], the plastron of *Podocnemis expansa* [11], the carpal and tarsal bones of several species [12], and the metacarpals and phalanges of *P. expansa* [13,14]. The state of knowledge is more limited for recently described species, such as the Orinoco Matamata turtle, *Chelus orinocensis* (Chelidae), for which no anatomical description is currently available. This species was described in 2020 by Vargas et al [15], who conducted a molecular and morphological analysis that confirmed the existence of two evolutionary lines that diverged approximately 12.7 million years ago: The Amazon Matamata turtle *Chelus fimbriata*, distributed in the Amazon and the Mahury river basins, and the Orinoco Matamata turtle, *Chelus orinocensis*, found in the Orinoco, Negro, and Essequibo rivers of northern South America.

The intensive illegal trade of matamata turtles represents perhaps the most serious threat to the species, particularly for hatchlings and juveniles, which are sold as pets in national and international markets [16,17]. To a lesser extent, matamatas are also captured for human consumption and used in the traditional medicine by some indigenous communities [18]. Although *C. orinocensis* is not currently considered endangered by the International Union for Conservation of Nature (IUCN) and it is listed as Low Concern (LC) [19], it is included in Appendix II of the Convention on International Trade in Endangered Species of Wild Fauna and Flora (CITES) [19], which regulates its international trade.

Knowledge of the basic anatomy of animals is essential for understanding how their bodies function and for advancing research in biology, natural history, and veterinary medicine. In particular, the study of the skeletal system enables the description of the shape, size, and arrangement of structural components, and allows inferences about ossification processes during early developmental stages [20,21]. In this context, the main objective of the present study was to describe and illustrate, through photographs, the morphology of the skeletal system of *Chelus orinocensis* neonates, including the cranium, mandible, hyoid apparatus, and the axial and appendicular skeletons.

## Materials and methods

### Ethical considerations

This study (code 24-2-955) received expedited ethical approval from the Ethics Committee for Animal Experimentation (CICUA) of the University of Antioquia. This approval was recorded in Act 159 from June 18, 2024.

### Specimens used

In 2016, the environmental authority Corporación para el Desarrollo Sostenible del Sur de la Amazonía (Corpoamazonía) confiscated 551 matamata turtle neonates in the city of Leticia, Amazonas Department, Colombia [17]. To determine the geographic origin of surviving turtles for their release, the Alexander von Humboldt Biological Resources Research Institute and the Universidad de los Andes conducted molecular analyses on some of the deceased individuals. Such analyses confirmed that the confiscated turtles had been captured in the Orinoco river basin [17]. Later, in 2020, two divergent lineages of matamatas were described [15], confirming that the individuals from the Orinoco river basin corresponded to a new species, Chelu*s orinocensis*. From this confiscation, 91 deceased specimens were donated to the Universidad de Antioquia for research purposes. The turtles were preserved by freezing in the Animal Anatomy Laboratory and were subsequently used in this study.

### Skeletonization

To describe the morphology of the skeletal system, ten hatchlings were selected from the donated specimens, while the remaining individuals were used for other studies. These ten hatchlings were skeletonized, and the two largest specimens were disarticulated to allow a detailed morphological description of each bone. The specimens were initially thawed, after which all internal and external soft tissues were carefully removed using scalpels, scissors, and forceps until the bones were fully exposed. A 3% hydrogen peroxide solution was then applied to the entire skeleton, with particular attention to areas where soft tissue remained, to ensure complete dissolution [21,22]. The skeletons were allowed to dry in the sun for approximately three days. Then, the individual bones of the disarticulated skeletons were numbered, measured using a digital caliper, and stored on a tray for further identification with reference to published literature [5, 23]. All bones were photographed, with smaller bones imaged using a camera adapted to a stereoscope and larger bones with an iPhone 15 Pro Max.

## Results

**Fig 1:**
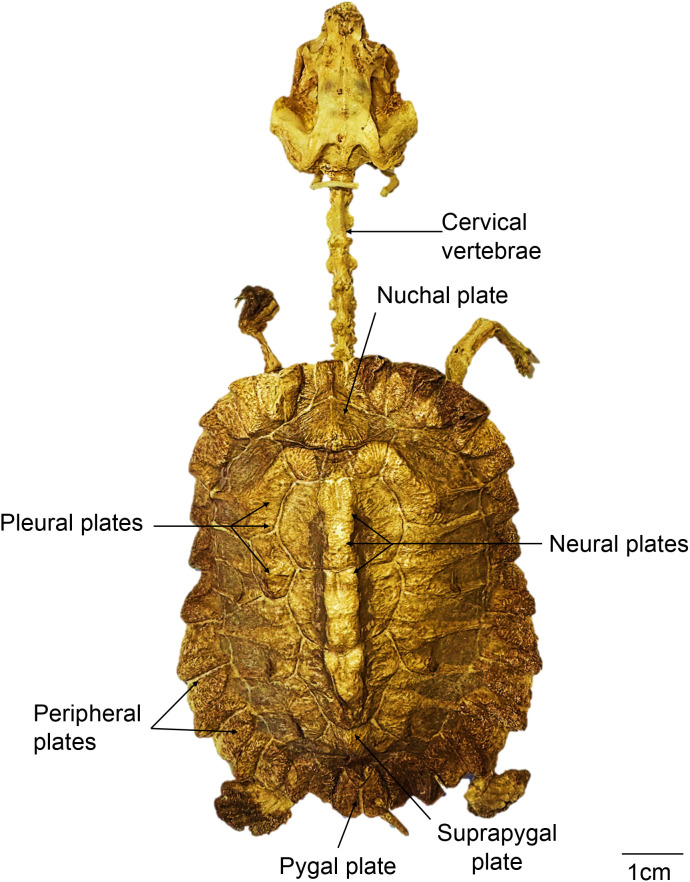
Complete articulated skeleton of a neonate of *Chelus orinocensis* (ID 13, Straight Carapace Length, SCL= 69.9 mm).

The group of ten turtles chosen for this study measured between 51.03 and 80.3 mm of straight carapace length (SCL, average = 69.56 mm), and the two turtles used for individual bone descriptions measured 72.58 and 80.3 mm of straight carapace length (SCL). The complete skeleton of these disarticulated individuals comprised a total of 288 bones each; however, three of the smallest individuals (which were not disarticulated) had 18 caudal vertebrae instead of 16 (Fig 1). S1 Table lists all observed bones, indicating whether they are pair or unpair, along with their linear measurements (S1 Table). The morphology of the skull, mandible, hyoid apparatus, and the axial and appendicular skeleton is described below.

### Skull

**Fig 2:**
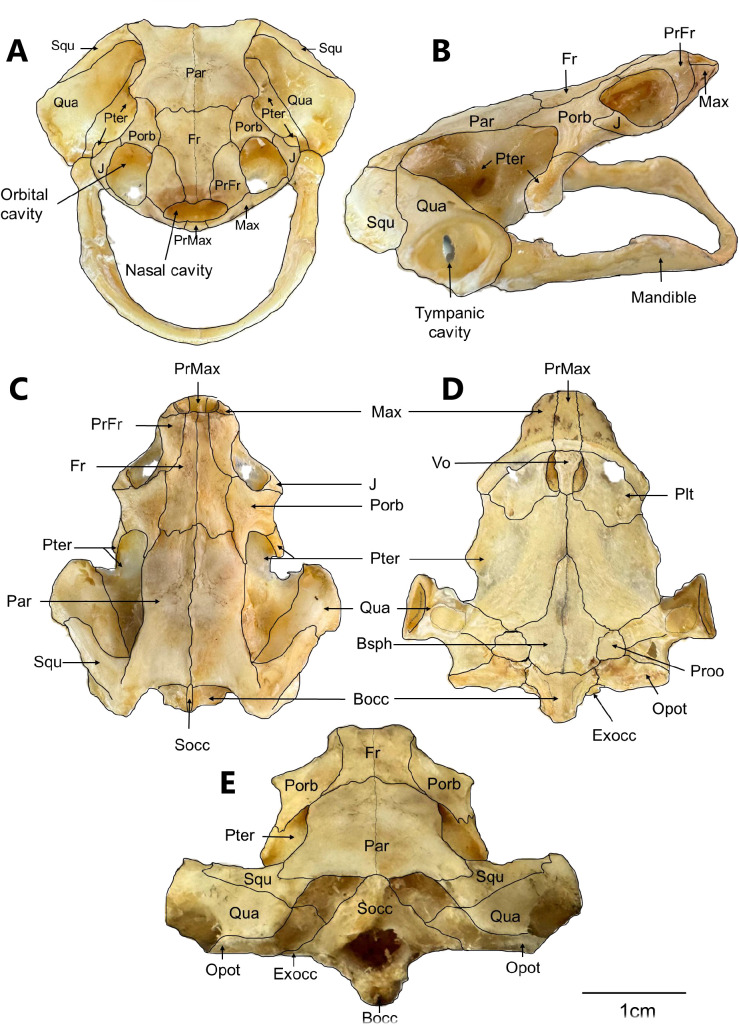
Skull of a *Chelus orinocensis* neonate (ID 13, SCL = 69.3 mm). A. rostral. B. lateral. **C.** dorsal **D.** ventral. E. caudal views of the skull. Bocc: basioccipital; Bsph: basisphenoid; Exocc: exoccipital; Fr: frontal; J: jugal; Max: maxilla; Opot: opisthotic; Par: parietal; Plt: palatine; Porb: postorbital; PrFr: prefrontal; PrMax: premaxilla; Proo: prootic; Pter: pterygoid; Qua: quadrate; Socc: supracoccipital; Squ: squamosal; Vo: vomer. Black lines were added to the figures to delineate the bone sutures.

The head is flattened and triangular, with length approximately equal to width. It lacks the nasal bone, a characteristic feature of the Chelidae family (Fig 2). The orbital cavity is relatively small, whereas the tympanic cavity is notably large compared with that of turtles in general. The individual cranial bones are described below in a caudorostral sequence from dorsal, ventral and lateral views, following the terminology of Gaffney [7,8] and Lemell et al [6].

**Occipital**. The occipital is an composite bone located in the caudal region of the skull. It forms the foramen magnum and part of the cranial roof. It articulates with the first cervical vertebra and is composed of three elements arranged dorsoventrally: the supraoccipital, exoccipital, and basioccipital (S1 Fig). The **supraoccipital** forms the caudal half of the cranial vault and thus the dorsal margin of the foramen magnum. It joins rostrally with the parietal, rostrolaterally with the opisthotic, and caudoventrally with the exoccipital. The supraoccipital process is absent. The paired **exoccipital** contributes to the lateral margins of the foramen magnum and to the dorsal portions of the occipital condyle. It contacts the supraoccipital dorsally, the basioccipital ventrally, and the opisthotic laterally. Two foramina for the hyploglossal nerve can be observed in this small bone. The **basioccipital** forms the base of the skull supporting the brainstem. It constitutes the ventral border of the foramen magnum and the ventral portion of the occipital condyle, which articulates with the first cervical vertebra.

**Basisphenoid**. The basisphenoid (S2 Fig.) is an unpaired, trapezoidal, flat bone with its base oriented caudally. It forms the floor of the cranial cavity and from a dorsal view it bears a central depression called the *Sella turcica*. It articulates cranially with the palatine, rostrolaterally with the pterygoid, caudolaterally with the prootic, and caudally with the basioccipital.

**Prootic**. The prootic bone (S3 Fig.) is a paired, irregularly shaped bone that contributes to both the cranial and otic chambers. It forms the anterior half of the floor of the temporal fossa and part of the lateral cranial wall. It contains several foramina for vessels and nerves, including the carotid, acoustic, facial, and trigeminal canals. Dorsally, it articulates medially with the supraoccipital, rostrodorsally with the parietal, rostrally with the pterygoid, laterally with the quadrate, and caudally with the opisthotic. Ventrally, it contacts the basisphenoid medially, the pterygoid rostrally, the quadrate laterally, and the opisthotic posteriorly.

**Opisthotic**. The opisthotic (S4 Fig.) is a paired, elongated bone that forms the caudal portion of the floor of the temporal fossa. It articulates dorsomedially with the supraoccipital, cranially with the prootic, laterally with the quadrate and squamosal, and ventrally with the basisphenoid and basioccipital. It bears a medial jugular foramen shaped like a broad, flat leaf with rounded margins, as well as a caudally directed paroccipital process.

**Quadrate**. The quadrate (S5 Fig.) is a paired, cylindrical bone that forms the posterolateral region of the skull. It projects laterally to form the auditory canal (*Cavum tympani*), overlies the temporal fossa, and bears a suboval opening that accommodates the tympanic membrane. The auditory canal is caudally divided by a notch, the *Columella auris,* and communicates posteriorly with the postotic antrum. The quadrate articulates cranially with the pterygoid, caudolaterally with the squamosal, medially with the opisthotic and prootic and ventrally with the articular bone of the mandible through a synovial articulation.

**Prefrontal**. The prefrontal (S6 Fig.) is a paired, narrow bone shaped like a pointed “J.” It forms the rostromedial border of the orbit and the dorsal margin of the nasal opening. It articulates rostrally with the maxilla and caudally with the frontal.

**Frontal**. The frontal (S7 Fig.) is a paired, elongated bone forming the central region of the rostral skull, including the caudomedial orbit, and extending to the dorsal margin of the nasal opening. The paired frontals meet at the midline and articulate caudally with the parietal, rostrolaterally with the prefrontal, and laterally with the postorbital.

**Parietal**. The parietal (S8 Fig.) is a paired, elongated, rectangular bone that forms most of the cranial roof and the lateral walls. The two parietals meet dorsally at the midline along the interparietal suture. Each parietal articulates cranially with the frontal, rostrolaterally with the postorbital, ventrocaudally with the prootic, ventrally with the pterygoid, and caudally with the supraoccipital.

**Postorbital**. The postorbital (S9 Fig.) is a paired, elongated with a caudally pointed bone. It contributes to the orbital roof, forms the rostrolateral border of the orbit with the jugal, articulates caudally with the pterygoid, and contacts the frontal and parietal medially and the prefrontal rostrally.

**Squamosal**. The squamosal (S10 Fig.) is a paired, thin bone that narrows distally, forming the caudal wall of the auditory canal (*Cavum tympani*) and the postotic antrum. A squamosal crest is present in the rostromedial region. It articulates rostromedially with the quadrate, caudomedially with the opisthotic, and medially with the parietal.

**Premaxilla**. The premaxilla (S11 Fig.) is an unpaired, cylindrical, conical bone with its narrowest part projected cranially. It forms the rostral part of the triturating surface and the ventral border of the nasal aperture. Laterally, it joins the maxilla to form the labial crest.

**Maxilla**. The maxilla (S12 Fig.) is a paired bone that forms the ventral margin of the orbit and most of the external arch of the upper jaw. It bears a prefrontal process that articulates with the prefrontal bone. It articulates caudomedially with the palatine and caudolaterally with the jugal.

**Jugal**. The jugal (S13 Fig.) is a paired, elongated bone forming the ventral margin of the orbit and the lateral wall posterior to the orbit. It articulates rostrally with the maxilla, medially with the palatine, dorsally with the postorbital, and caudally with the pterygoid.

**Vomer**. The vomer (S14 Fig.) is a small, flat, unpaired bone forming the medial margin of the choanae. It articulates rostrally with the maxillae, caudally with the pterygoids, and laterally with the palatines.

**Palatine**. The palatine (S15 Fig.) is a paired, thin, flat bone shaped like a scale. It forms the anterior third of the palate and, together with the vomer, borders the choanae laterally. It articulates rostrally with the maxilla and the jugal, and caudolaterally with the pterygoid.

**Pterygoid.** The pterygoids (S16 Fig.) are paired, elongated bones and the largest elements of the ventral skull. They contribute to the lateral portion of the cranium, and the medial part of the ear region. Each pterygoid bears a single trochlear process. The pterygoids articulate only at their anterior medial third and are separated caudally by the basisphenoid. Rostrolaterally, they articulate with the postorbital and jugal bones. From a ventral view, they articulate rostrally with the palatines, medially with the basisphenoid, caudally with the prootic, caudolaterally with the quadrate, and rostromedially with the vomer.

### Mandible

**Fig 3:**
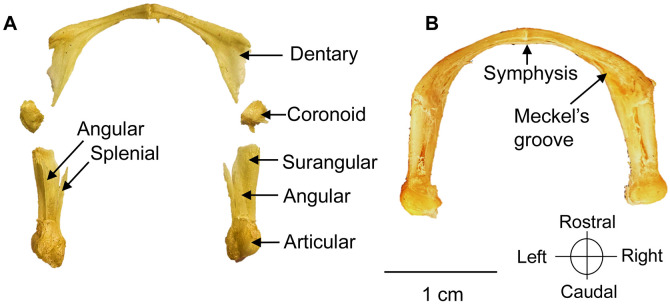
Mandible of a *Chelus orinocensis* neonate (ID 20, SCL = 80.3 mm). **A.** Mandible disassembled in dorsal view. **B.** Mandible complete in dorsal view. The angular bone fig 3A on the left side is detached.

The mandible of *C. orinocensis* is a slender, a U-shaped arch. It is composed of six paired bones (Fig 3).

**Dentary.** The dentary is the largest mandibular element, forming the rostral portion and constituting the mandibular symphysis. Its labial margin is more developed than the lingual, bearing a sharp cutting crest along the dorsal surface. It articulates with the coronoid, splenial, angular, and surangular. On its medial surface, it exhibits an elongated concavity, the Meckel’s groove, which continues into the adductor (Meckelian) fossa.

**Coronoid.** The coronoid is the smallest mandibular element, with a semicircular, irregular outline. Together with the dentary, it contributes to the dorsolateral coronoid process and the adductor fossa in the mid-mandibular region. It articulates rostrally with the dentary and caudally with the angular, surangular and splenial.

**Surangular**. The surangular is the most developed bone of the caudolateral mandibular region. It forms the posterolateral portion of each hemimandible and the lateral wall of the adductor fossa. A robust rostral process projects towards the labial surface of the dentary, reaching the coronoid process. It articulates dorsally with the coronoid, rostrally with the dentary, ventrally with the angular, and caudally with the articular.

**Angular.** The angular is an elongated, slender bone located medially. It articulates rostrally the dentary and coronoid, dorsolaterally the splenial and surangular, and caudally the articular.

**Splenial.** The splenial is an elongated bone positioned centrally on the medial surface of each hemimandible. It forms part of the floor of the adductor fossa and the lateral wall of Meckel’s groove. Dorsally, it lies between the angular and surangular.

**Articular.** The articular is the most caudal mandibular element, with a semicircular shape. It bears a convex articular surface, which articulates with the quadrate, forming a loose joint. This articulation permits a wide gape, characteristic of pleurodires. It articulates the surangular rostrolaterally, the splenial rostromedially, and the angular ventrally.

### Hyoids

**Fig 4:**
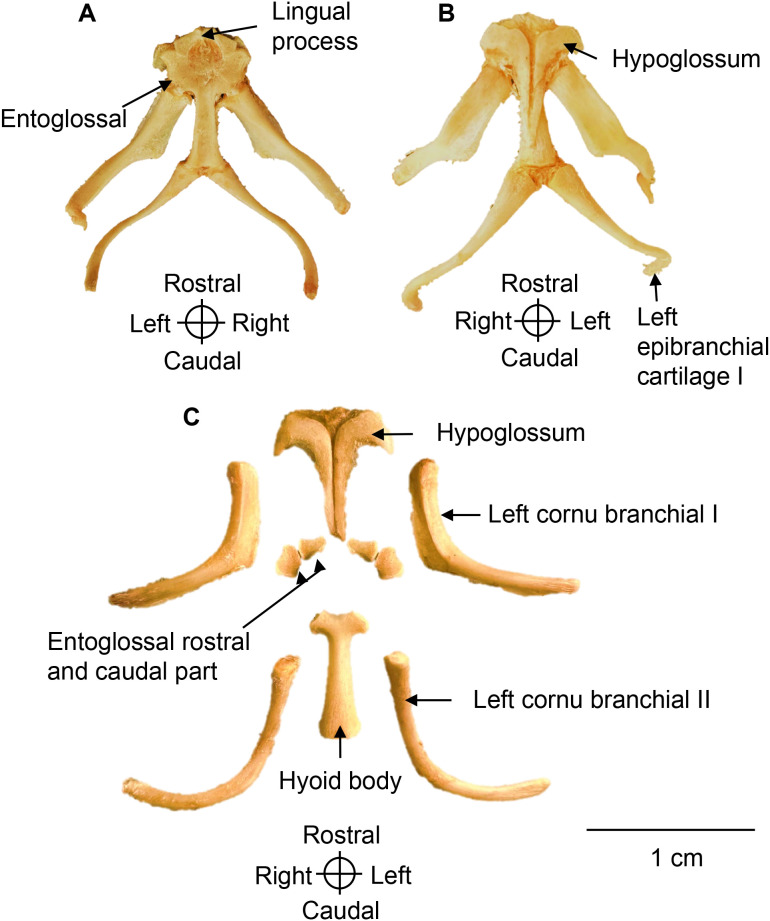
Hyoid apparatus of a *Chelus orinocensis* neonate. **A.** Hyoid apparatus in dorsal view, **B.** Ventral view in individual ID 20 (SCL = 80.3 mm). **C.** Hyoid apparatus disassembled in individual ID 11 (SCL = 72.58 mm) in ventral view to expose each bone individually.

The hyoid apparatus is large, measuring approximately two-thirds the length of the skull. It is fully ossified, except for the epibranchial I and II cartilages and the lingual process (Fig 4). In the five larger specimens examined, the epibranchial I cartilage was partially ossified.

**Hypoglossum.** The hypoglossum is a large, flat, unpaired bone located ventrorostral to the hyoid and unique to Testudines. It is anchor-shaped, comprising two bony bars that are firmly fused along the midline. The caudal portion is narrow and tapered, whereas the rostral portion expands laterally into horn-like projections. The rostral margin continues into hypoglossal cartilage.

**Entoglossal.** The entoglossal are small, paired, and semicircular bones that surround the lingual cartilage. They are positioned rostral to the hyoid body and dorsal to the hypoglossum, without contacting one another at the midline. In the neonates examined, each entoglossal was divided into a cranial and a caudal segment, although it is unclear whether this condition persists in larger individuals.

Hyoid body. The hyoid is a single median bone that widens rostrally to form a cartilaginous lingual process and extends caudally as the tracheal groove. It articulates with two pairs of branchial horns: the *cornu branchiale* I (rostrolateral) and the *cornu branchiale* II (caudolateral).

***Cornu branchiale* I**. The *cornu branchiale* I is a robust paired bone that bears, at its distal end, a small cartilaginous bar (the epibranchial I) which curves medially. In individuals larger than 72.8 mm SSL, these cartilages exhibited partial ossification. The proximal portion of each horn is horizontally flattened and extends to the caudal margin of the tracheal groove of the hyoid body.

***Cornu branchiale* II.** The *cornu branchiale* II is also a paired bone, that is longer, thinner, and more oblique than *cornu branchiale* I. These elements exhibit a pronounced curvature, with their distal ends directed medially and positioned caudal to the skull. Proximally, they articulate with the caudal region of the hyoid body, and distally they support the epibranchial II cartilage.

### Axial skeleton

**Fig 5:**
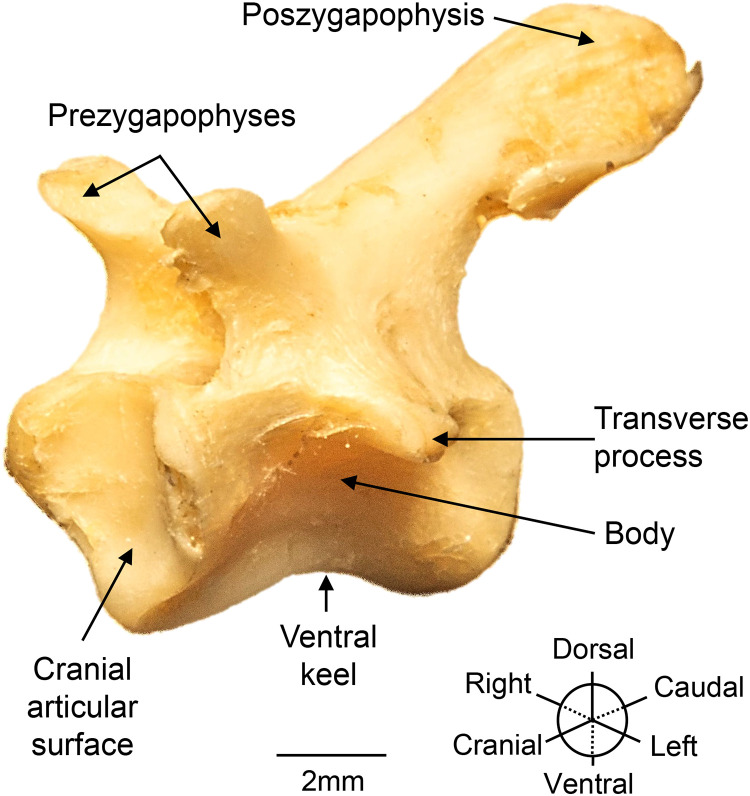
Structures of cervical vertebra 7 (C7) of a *Chelus orinocensis* neonate (ID 20, SCL = 80.3 mm), oblique view.

**Fig 6:**
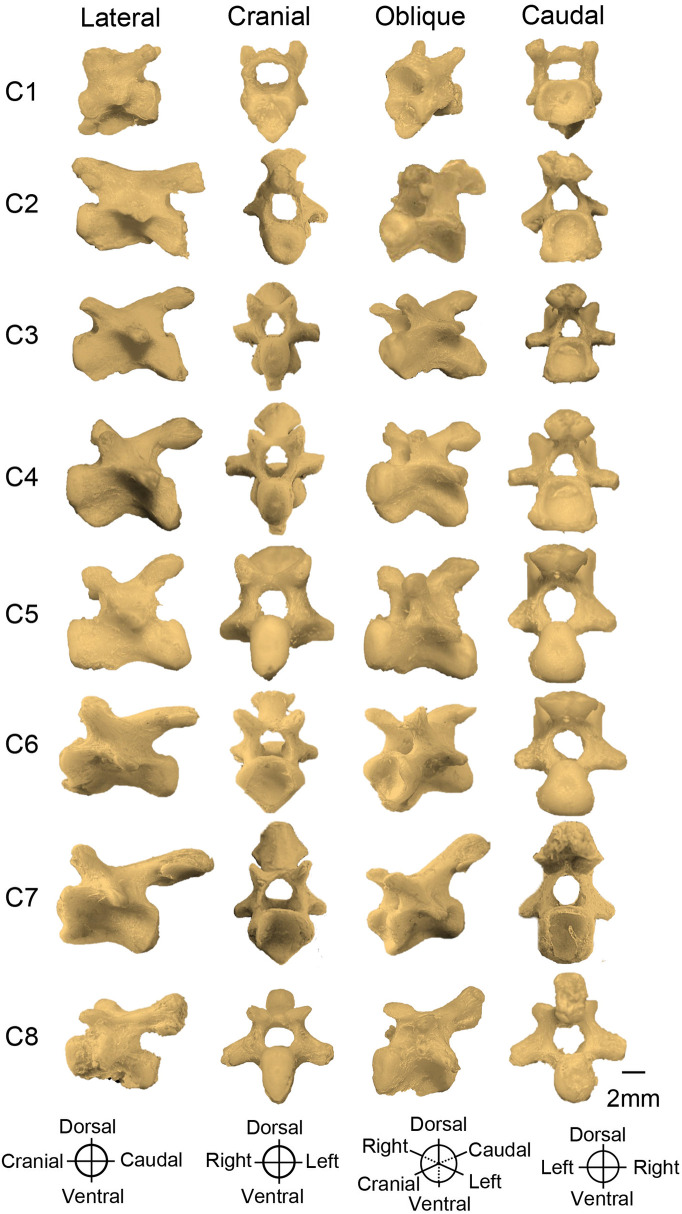
Cervical vertebrae of a *Chelus orinocensis* neonate in lateral, cranial, oblique and caudal views.

The vertebral column of *Chelus orinocensis* consists of 8 cervical, 10 dorsal, 2 sacral, and 16–18 caudal vertebrae (vertebral formula = C8, D10, S2, Ca16–18) (Figs 5, 6).

**Cervical Vertebrae.** C1 is the smallest of the eight cervical vertebrae (Fig 6). It possesses an amphicoelous (biconcave) centrum and articulates cranially with the occipital bones. It lacks prezygapophyses and bears a widely bifurcated postzygapophyses that articulates with C2. The transverse processes are small and project caudoventrally. From the dorsal surface of its centrum, a pair of arches arises and fuses at the midline to form the vertebral foramen.

Vertebrae C2-C4 have longer centra than C1, are opisthocoelous (cranially convex, caudally concave), have a ventral keel, and possess well-developed transverse processes. Their postzygapophyses are unforked, whereas their prezygapophyses are bifid, except in C2 that is non-bifid.

Vertebra C5 is biconvex, with a narrower and smaller centra than C2 and C7 but retain a ventral keel and prominent transverse processes. The articular facets on the postzygapophyses increase progressively in size from C1 to C7 and decrease again at C8.

C6 is procoelous (cranially concave, caudally convex). C7 is amphicoelous, bears a ventral keel, and the most strongly developed postzygapophyses and articular facets within the cervical series. C8 is biconvex, with a short centrum and the most prominent ventral keel. It possesses a large postzygapophysis that articulates with Dorsal 1 (D1), although its articular facets are less developed than those of the more cranial cervical vertebrae.

**Dorsal vertebrae.** *C. orinocensis* possesses 10 dorsal vertebrae, as is typical for Testudines. These vertebrae are articulated to one another, and to the rib heads. The neural arches of dorsal vertebrae expand dorsally to form the neural plates in an intercentral arrangement; that is, a single neural plate may cover two or more vertebrae depending on its position (dorsal vertebrae decrease in size toward the caudal end). D2 to D8 articulate with ribs 2–8 in an intercentral configuration, meaning that each rib is articulated to two consecutive vertebral bodies.

Vertebra D1 is procoelous, with prezygapophyses oriented forward to articulate with the postzygapophyses of C8. Its neural arch extend to the first neural bone, and its transverse processes bear the first pair of ribs. Vertebrae D2 to D10 are platycoelous, with flat articular surfaces. From D2 to D8, the vertebral bodies are narrow and elongated, decreasing in size caudally. D8 is the shortest and least compressed vertebra of the dorsal series. D9 and D10 are short, with small transverse processes that articulate with ribs 9 and 10, respectively.

**Sacral vertebrae.** The sacral region comprises two vertebrae, which are not fused to the carapace. The transverse processes of S1 articulate with the ilium via short cartilaginous ribs. On the other hand, S2 possesses a caudal condyle that articulates with Ca1.

**Caudal vertebrae.** The tail comprises 16–18 procoelous caudal vertebrae, which progressively decrease in thickness and height toward the caudal end due to a reduction of the neural arch size. Ca1 to Ca4 are the tallest vertebrae, with neural arches more developed than their centra and bearing a short neural crest. From Ca5 to the terminal vertebra, the vertebrae are shorter and lack neural arches. Prezygapophyses and postzygapophyses are present but weakly developed, gradually decreasing in size toward the tail tip.

### Carapace

**Fig 7:**
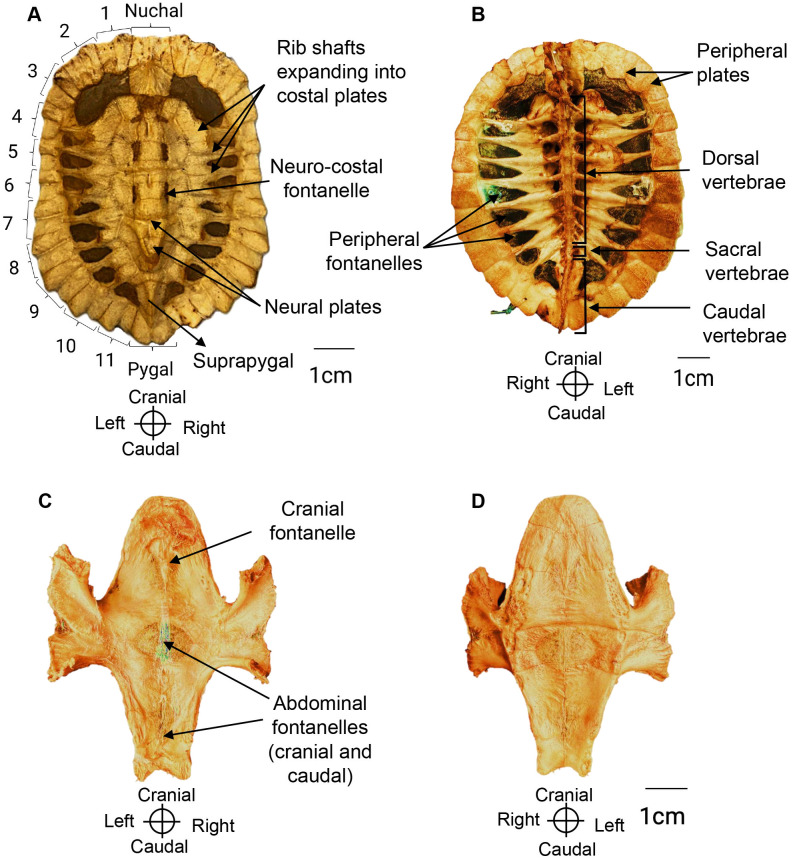
Carapace and plastron of *Chelus orinocensis* neonates. **A.**Carapace in dorsal view indicating the dermal bones in individual ID 63 (SCL = 76.07 mm). **B.** Carapace in ventral view in individual ID 11 (SCL = 72.58 mm). C, **D.** Plastron in dorsal and ventral views respectively in individual ID 11. Incomplete growth of dermal bones results in the presence of fontanelles in carapace and plastron.

The carapace is covered externally by a total of 34 keratinized scutes: one nuchal, five vertebral, four pairs of costal, and 12 pairs of marginal scutes. More internally there are the dorsal vertebrae (described above), the ribs, and their respective dermal bones (Fig 7).

**Ribs**. Ten ribs were observed. Rib 1 is short, but still in contact with the first pleural plate and caudally with rib 2. Ribs 2–9 suture medially at the junction of two consecutive vertebral bodies. They grow laterally until their distal ends connect with the peripheral plates. Rib 10 is small and, together with rib 9, fuses to the eighth pleural plate. Ribs also expand in cranio-distal direction to form the pleural plates as described below.

**Fig 8:**
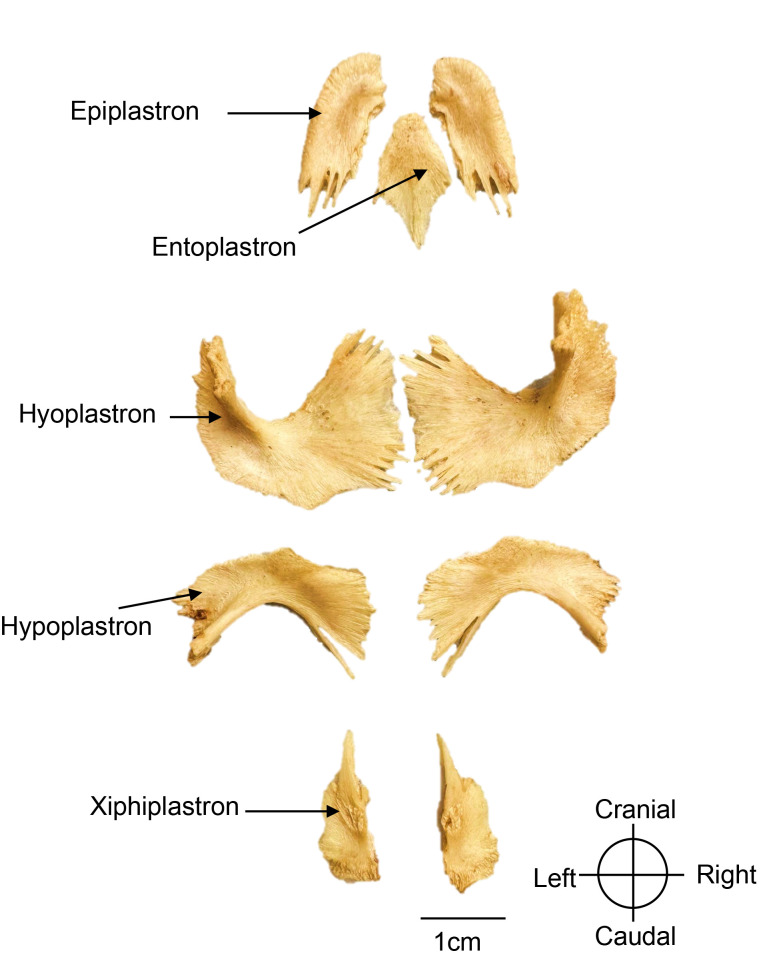
Dermal bones of the plastron disassembled in a *Chelus orinocensis* neonate (ID 20, SCL = 80.3 mm), in dorsal view.

**Dermal bones.** Along the midline, from cranial to caudal, the dermal bones comprise one nuchal plate, seven neural plates, and one suprapygal plate (Figs 7, 8). Also, on each side, there are eight pairs of pleural plates, bordered by 11 peripheral plates per side, and one pygal plate. The nuchal plate is single, and rectangular, and does not contact any vertebrae. The neural plates develop dorsally as extensions of the vertebrae. They were in contact to one another along the midline, and then they extend laterally to meet the pleural plates. The suprapygal plate is elongated and represents the most caudal midline bone; and like the nuchal, it does not contact any vertebra or rib. The pleural plates overlie ribs 2–9, and their expansion varied with the individual body size. The smallest individual reviewed (ID 84, straight carapace length, CL = 55.42 mm) had small pleural plates not in touch with the neural plates, i.e., leaving pleural fontanelles open (i.e., on each side of the vertebrae), nor with the peripheral plates leaving peripheral fontanelles open. On the other side, the larger individuals (ID 65, SCL = 78.21 mm) had larger pleural plates such that the vertebral fontanelles were closed but the peripheral fontanelles were still open. In all cases, the distal portions of the ribs remained exposed. The peripheral plates also develop posthatching and receive the distal tip of the ribs, but is not a 1:1 ratio, as ribs 2–9 fit into plates 4–10 (8 ribs for 7 plates). Finally, the pygal plate is the posterior most plate along the midline.

### Plastron

The plastron forms the ventral shell. It is attached via ligaments to the pectoral girdle and sutured to the pelvic girdle as in other pleurodires (side-necked turtles). Beneath the epidermis, dermal bones constitute plastral bones (Figs 7, 8). In neonates (SSL = 80 mm), these bones are incompletely fused, leaving broad midline fontanelles that allow for continued growth.

**Fig 9:**
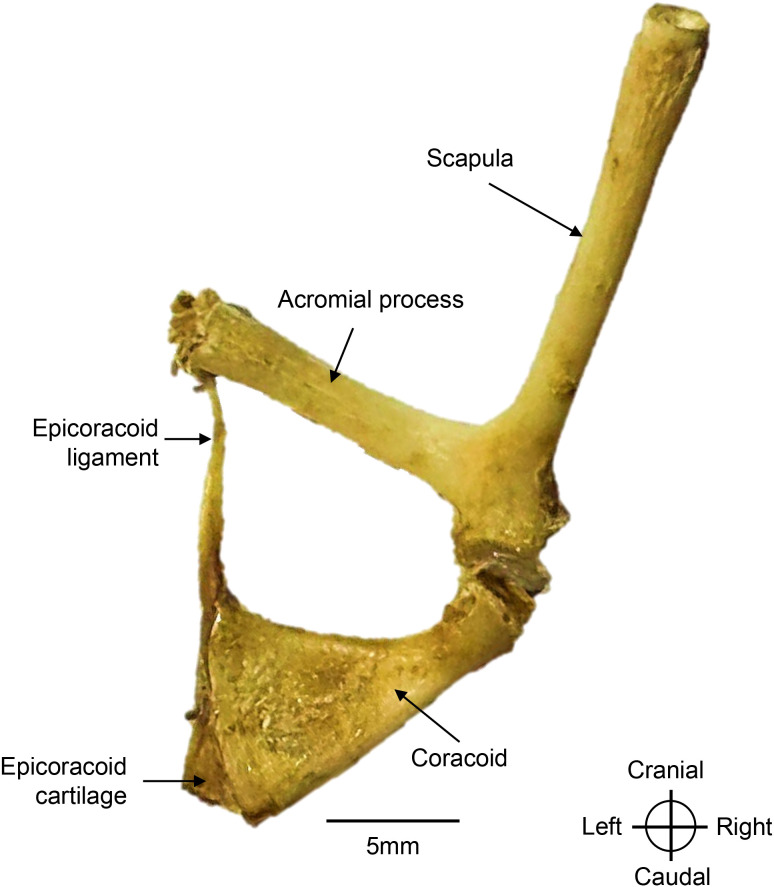
Right pectoral girdle of a *Chelus orinocensis* neonate (ID 20, SCL = 80.3 mm), shown in dorsal view.

The plastron consists of nine dermal bones under the epidermal keratinous scutes, that are larger and therefore do not align perfectly with the underlying bones. Although these scutes were removed, their impressions remain on the dermal bones at this early developmental stage. From cranial to caudal, the dermal bones are the paired epiplastra, the single entoplastron, and the paired hyoplastra, hypoplastra, and xiphiplastra (Fig 9). The two epiplastra meet medially, and their finger-like projections extending caudally along both sides of the entoplastron. The entoplastron is rhomboid in shape and partially fills the cranial fontanelle in neonates (Fig 7). The hyoplastra and hypoplastra interlock medially but leave an open space corresponding to the abdominal fontanelle. They form the axillary and inguinal pillars, respectively, which support the bridge. These pillars articulate laterally with ribs 2–6 and peripheral plates 4–7, forming a robust junction with the carapace. Finally, the xiphiplastra bear strong medial projections that suture anteriorly with the hypoplastra and dorsally with the pubis and ischium, reinforcing the connection to the pelvic girdle.

## Appendicular skeleton

### Pectoral girdle

The pectoral girdle exhibits a triradiate morphology and is composed of two bones: an elongated scapula with its acromial process, and the coracoid bone (Fig 9). The scapula is oriented dorsomedially, with the acromial process extending ventromedially, while the coracoid is oriented caudoventrally toward the midline.

**Scapula**. The scapula is the longest bone of the pectoral girdle. Dorsally, it contacts the internal surface of the carapace via ligaments at the first pleural plate, and distally it articulates with the coracoid at right angle to form the glenoid fossa, which in turn articulates with the humerus. The scapula bears a large acromial process that extends over more than half its length. This acromial process is firmly attached to the entoplastron via ligaments, thus anchoring the pectoral girdle to the plastron.

**Coracoid**. The coracoid is a broad, flat, fan-shaped bone. Its widest margin attaches firmly to the hyoplastron at an approximate 45° angle. The distal border of the coracoid bears an epicoracoid cartilage, from which a ligament originates and extends to the acromial process.

### Forelimb

**Fig 10:**
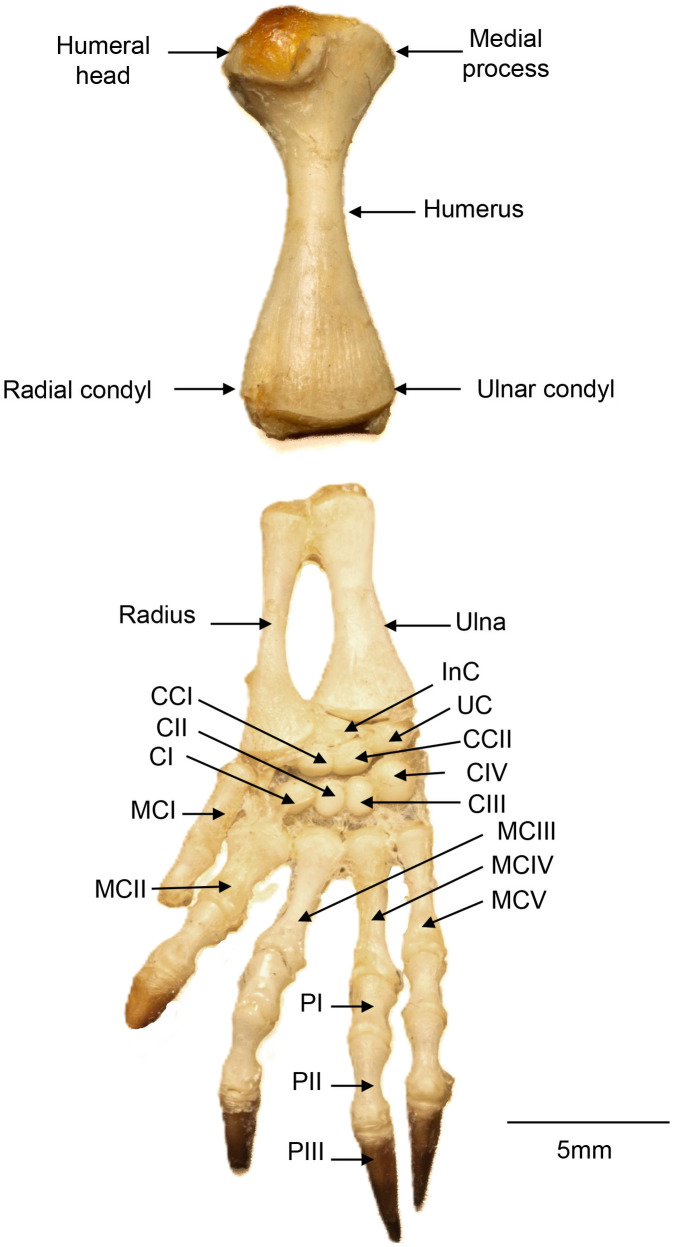
Forelimb (left side) of *Chelus orinocensis* neonate (ID 20, SCL = 80.3 mm, shown in dorsal view. CCI: central carpal I; CCII: central carpal II; CI: carpal I; CII: carpal II; CIII: carpal III; CIV: carpal IV; InC: intermediate carpal; MCI: metacarpal I; MCII: metacarpal II; MCIII: metacarpal III; MCIV: metacarpal IV; MCV: metacarpal V; PI: phalange I; PII: phalange II; PIII: phalange III; UC: ulnar carpal. Digit 1 is missing the distal phalange, digit 2 is missing its claw.

The forelimb is composed of appendicular bones, including the humerus, radius, ulna, carpals, metacarpals, and phalanges (Fig 10).

**Humerus**. Proximally, the humerus articulates with the glenoid fossa built by scapula and coracoid, and distally with the radius and ulna. The proximal epiphysis bears a well-developed central head, a wide intertubercular fossa, and two processes: a larger lateral projection and a smaller medial projection. The humerus shaft narrows at midlength and expands slightly distally, forming a craniocaudally flattened region that articulates with the radius and ulna.

**Radius and Ulna.** The radius is positioned medially in the forelimb and is more elongated and slender than the ulna (Fig 10). Its distal epiphysis expands to articulate with the proximal, middle, and distal rows of carpal bones. The ulna lies laterally and is more robust but shorter than the radius. Its distal epiphysis articulates with the proximal row of carpal bones.

**Carpals.** Carpal bones are arranged in three rows (Fig 10). The proximal row includes the ulnar carpal laterally, and the intermediate carpal medially. The middle row consists of central carpals I and II. The distal row comprises four carpals, numbered medially to laterally as CI, CII, CIII, and CIV. The pisiform and accessory bones, which are usually located near the radius and are typically the last to ossify, were not observed in the neonate secimens examined.

**Metacarpals.**. The metacarpals comprise five elongated bones, numbered medially to laterally as MCI, MCII, MCIII, MCIV, and MCV (Fig 10). Each has a proximal base that articulates with the distal row of carpal bones and a distal head that articulates with the proximal phalanges.

**Phalanges**. The manus comprises five digits. The first digit is the smallest, with only two phalanges (proximal and distal), whereas the remaining four digits each have three phalanges (proximal, middle and distal). Accordingly, the phalangeal formula of the manus, from medial to lateral, is 2-3-3-3-3 (Fig 10). The middle phalanges are the longest, while the distal phalanges are the shortest and bear a conical tip to support the keratinous claw sheath.

### Pelvic Girdle

**Fig 11:**
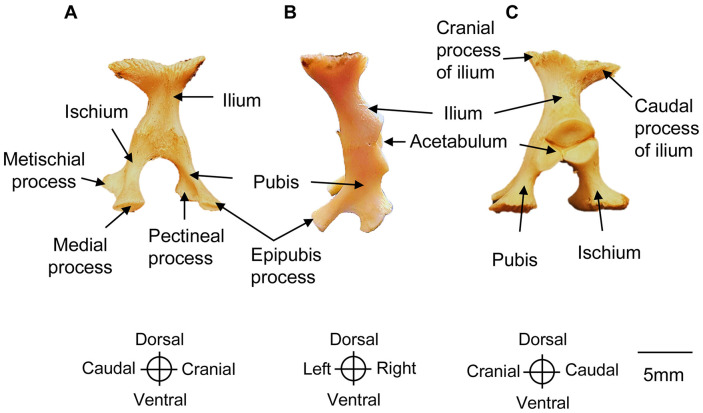
Pelvic girdle (left side) of a *Chelus orinocensis* neonate (ID 20, SCL = 80.3 mm). A. Medial view, B. Cranial view, C. Lateral view.

The pelvic girdle is formed by three bones: ilium, ischium, and pubis (Fig 11).

**Ilium.** The ilium is a paired, thick, columnar bone extending dorsally from the acetabulum. It expands dorsally into a broad rugose surface with cranial and caudal processes that fuse with the last pleural plate and the suprapygal bone of the carapace respectively. Distally, its lateral margin contributes to the acetabulum, a concave surface shared with the ischium and pubis, which articulates with the femur at a right angle to facilitate the locomotion.

**Ischium.** The ischium is a paired bone projecting caudoventrally from the acetabulum to the caudal margin of the xiphiplastron, to which it is firmly sutured. A narrow medial process extends along the caudal edge of the xiphiplastron to the midline, reinforcing the connection with the plastron. It also bears a short but robust metischial process that projects caudolaterally toward the posterior tip of the xiphiplastron.

**Pubis.** The pubis extends cranioventrally from the acetabulum to the xiphiplastron, forming a weak suture at the pectineal process. The distal third of the pubis bears the epipubic process, a thin projection directed craniomedially toward the midline, but it does not contact the plastron.

### Hind Limb

**Fig 12:**
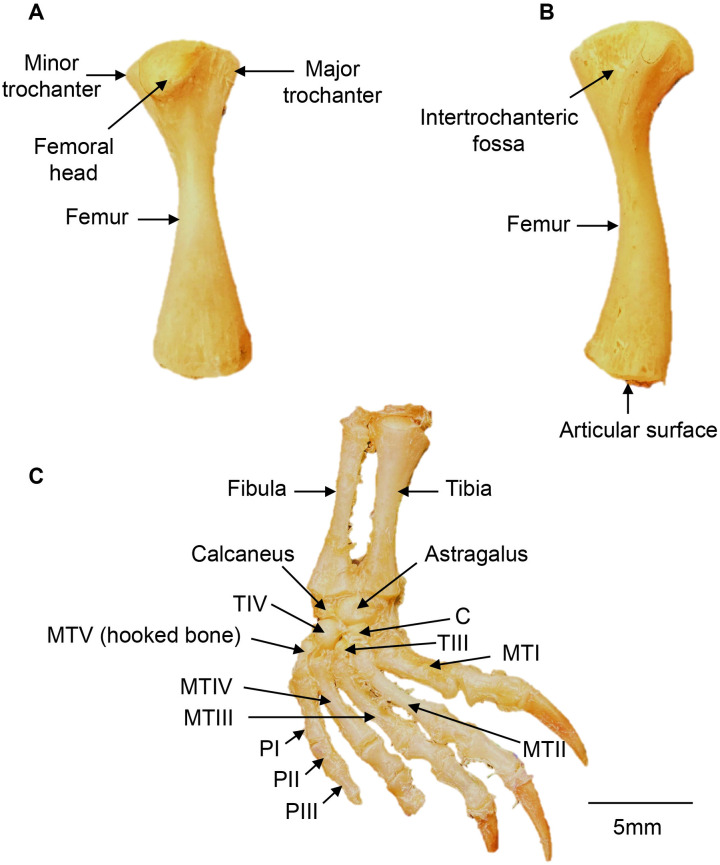
Hindlimb (right side) of a *Chelus orinocensis* neonate (ID 20, SCL = 80.3 mm). A. Femur in cranial view, B. Femur in caudal view, C. Distal hindlimb in dorsal view. C: central tarsal TIII: tarsal III; TIV: tarsal IV; MTI: metatarsal I; MTII: metatarsal II; MTIII: metatarsal III; MTIV: metatarsal IV; MTV: metatarsal V (hooked bone); PI: phalange I; PII: phalange II; PIII: phalange III. Digit 4 is missing the distal phalange and its claw.

The hind limb comprises the appendicular bones, including femur, tibia, fibula, tarsals, metatarsals, and phalanges (Fig 12).

**Femur.** The femur is the longest appendicular bone (Fig 12). It articulates proximally with the acetabulum and distally with the tibia and fibula via a single oval articular surface. The proximal epiphysis bears a cranially oriented head with two trochanters: the greater trochanter projects dorsally, and the lesser trochanter projects ventrally. Between them, on the caudal surface, lies a wide, shallow intertrochanteric fossa. The femoral shaft is narrow proximally and widens distally.

**Tibia and Fibula.** The tibia and fibula are two elongated bones that articulate proximally with the femur and distally with the proximal row of the tarsal bones. Both bones are similar in shape and size, although the tibia is slightly more robust. Their epiphyses are modestly expanded, and while both articulate distally with the calcaneus, only the fibula articulates with the astragalus.

**Tarsals.** Tarsals are arranged in three rows. In the neonates examined, the proximal row includes the astragalus, a large positioned medial bone that articulates medially with the tibia and laterally with the fibula; and the smaller calcaneus, which articulates with the fibula. The medial row contains the central tarsus, ventral to the astragalus; and the distal row, from medial to lateral, comprises tarsals TIII and TIV. The tarsals TI and TII are last to ossify and were not observed in the neonate specimens examined (Fig 12).

**Metatarsals.** The metatarsal comprises five elongated bones, numbered medially to laterally as MTI, MTII, MTIII, MTIV, and MTV. Each has a proximal base that articulates with the distal row of the tarsal bones and a distal head that articulates with the proximal phalanges. MTI is slightly shorter and broader than MTII-MTIV. MTV, also referred to as the “hooked bone”, is irregular and hook-shaped, positioned laterally at the level of the distal tarsal row, and articulates with the fifth digit (Fig 12).

**Phalanges.** As in the manus, the pes has five digits. The first digit has two phalanges and digits 2−5 have three phalanges each, giving a phalangeal formula: 2-3-3-3-3. The distal phalanges are conical, supporting the keratinous claw sheath, except digit 5 that does not have claw (Fig 12).

## Discussion

This study is the first to describe and photograph the complete skeleton of *Chelus orinocensis*. The specimens used in this study (n = 10) were small, with straight carapace lengths (SCL) ranging from 5.1 to 8.3 cm, and only the smallest specimen (SCL = 5.1 cm) retained an umbilical scar. Considering that Metrailler and Le Gratiet [24] reported *Chelus fimbriata* hatchlings in captivity reaching an SCL of 8 cm at six months of age, the specimens in this study can be considered between neonates to young juveniles under six months of age. At this developmental stage, we observed several notable skeletal characteristics: some bones were absent, others were incompletely ossified, and certain bones showed variation among the specimens examined.

Absent bones include the nasal, and the mesoplastron. The absence of the nasal is a character of *Chelus,* while the mesoplastron is a character of Chelidae [5] and some extant pleurodirans as well as fossil taxa [4].

Bones that were not fully ossified include the dermal bones of the carapace and plastron, resulting in fontanelles of varying sizes in the neonates examined. In *Chelus* sp, fontanelles persist until a carapace length of 15–20 cm [25]. In other species, such as *Emys orbicularis*, complete ossification of the carapace requires at least five years [26]. The hyoid was fully ossified except for the epibranchial cartilages I and II. In individuals larger than 72 mm SCL, incipient ossification was observed in epibranchial cartilage I, suggesting a progressive ossification in this region, consistent with previous reports in *Chelus fimbriata* and *Phrynops hilarii* [5,6]. Finally, the pisiform and accessory bones in the forelimb and the tarsals I and II bones in the hindlimb, were absent in this study, however they are present in *Chelus fimbriata* adults (Figs 2 and 8 in Sánchez-Villagra et al [27]), suggesting that these two bones may also ossify later in life in *C. orinocensis*.

Other bones showed notable variability among the specimens examined, including features not previously described in other turtle species, such as the segmentation of the entoglossal bones and the presence or absence of a fourth phalange in the pes. The entoglossal bones exhibited cranial and caudal segmentation on both the right and left sides. This condition may represent an early ontogenetic feature or a persistent trait of *Chelus orinocensis*, pending confirmation in adult specimens. Such segmentation has not been reported in the ontogenetic development of other turtles, including *Phrynops hilarii* [5], despite belonging to the same family.

Finally, some structures were underdeveloped such as the premaxilla, or highly developed, such as cervical vertebra 7 and 8, the hyoid apparatus, and the quadrate bone that forms the auditory canal. The reduction of the premaxilla may be explained by the suction feeder mechanism described for *Chelus, Chelonida* and *Hydromedusa* that benefits from having a triangular skull needed for a more efficient head forward thrust [6]. On the other side, the pronounced development of the postzygapophyses in C7 and C8 allows efficient neck flexion, potentially associated with the feeding mechanism, which involves a rapid whip-like strike towards the prey followed by suction, as described in *C. fimbriata* [6,28]. The elongations of neural spines of C7, and a little shorter in C8 of *Chelus orinocensis*, are shared with the extinct *Chelus colombiana*, but not with extant *Chelus fimbriata* [29]. On the other hand, the sacralization of dorsal vertebrae D9 and D10, positioned at the level of the ilium, is a typical pleurodiran feature that provides lumbosacral stability and support the body [22].

In this study we examined the complete skeleton of a rare turtle species for which little anatomical information is available. We opportunistically analyzed neonates confiscated from the illegal trade and provide detailed photographs, which are particularly useful and commonly referenced in the anatomical literature [7,8]. This study however is limited to the osseous development reached at this early life age. Further ontogenetic studies focusing on ossification in older individuals are essential to fully understand the evolutionary history and functional adaptations of this species.

## Supporting information

S1 Fig Occipital bone of the skull a *Chelus orinocensis* neonate in dorsal, caudal and lateral views. Black lines were added to delineate the bone sutures.
(JPG)

S2 Fig Basisphenoid bone of the skull of a *Chelus orinocensis* neonate. (JPG)

S3 Fig Prootic bone of the skull of a *Chelus orinocensis* neonate. (JPG)

S4 Fig Opisthotic bone of the skull of a *Chelus orinocensis* neonate. (JPG)

S5 Fig Quadrate bone of the skull of a *Chelus orinocensis* neonate. (JPG)

S6 Fig Prefrontal bone of the skull of a *Chelus orinocensis* neonate. (JPG)

S7 Fig Frontal bone of the skull of a *Chelus orinocensis* neonate. (JPG)

S8 Fig Parietal bone of the skull of a *Chelus orinocensis* neonate. (JPG)

S9 Fig Postorbital bone of the skull of a *Chelus orinocensis* neonate. (JPG)

S10 Fig Squamosal bone of the skull of a *Chelus orinocensis* neonate. (JPG)

S11 Fig Premaxilla bone of the skull of a *Chelus orinocensis* neonate. (JPG)

S12 Fig Maxilla bone of the skull of a *Chelus orinocensis* neonate. (JPG)

S13 Fig Jugal bone of the skull of a *Chelus orinocensis* neonate. (JPG)

S14 Fig Vomer bone of the skull of a *Chelus orinocensis* neonate. (JPG)

S15 Fig Palatine bone of the skull of a *Chelus orinocensis* neonate. (JPG)

S16 Fig Pterygoid bone of the skull of a Chelus orinocensis neonate. (JPG)

S1 Table List of observed bones of a *Chelus orinocensis* neonate, classification as paired or unpaired, and linear measurements. (DOCX)

## References

[1] Sheil CA. Osteology and skeletal development of Apalone spinifera (Reptilia: Testudines: Trionychidae). J Morphol. 2003;256(1):42–78. doi: 10.1002/jmor.10074 12616574

[2] Sheil CA. Skeletal development of Macrochelys temminckii (Reptilia: Testudines: Chelydridae). J Morphol. 2005;263(1):71–106. doi: 10.1002/jmor.10290 15536645

[3] Rieppel O. Studies on skeleton formation in reptiles: Patterns of ossification in the skeleton of Chelydra serpentina (Reptilia, Testudines). J Zool. 1993;231(3):487–509. doi: 10.1111/j.1469-7998.1993.tb01933.x

[4] Meylan PA. Skeletal morphology and relationships of the early cretaceous side-necked turtle, Araripemys barretoi (testudines: Pelomedusoides: Araripemydidae), from the santana formation of Brazil. J Vertebr Paleontol. 1996;16(1):20–33.

[5] Bona P, Alcalde L. Chondrocranium and skeletal development of Phrynops hilarii (Pleurodira: Chelidae). Acta Zoologica. 2009;90(4):301–25. doi: 10.1111/j.1463-6395.2008.00356.x

[6] Gumpenberger M, Gemel R, Lemell P, Snelderwaard P, Weisgram J, Beisser CJ. The feeding apparatus of Chelus fimbriatus (Pleurodira; Chelidae) – adaptation perfected?. Amphib Reptilia. 2010;31(1):97–107. doi: 10.1163/156853810790457803

[7] Gaffney ES. The side-necked turtle family Chelidae: a theory of relationships using shared derived characters. American Museum Novitates. 1977.

[8] Gaffney ES. Comparative cranial morphology of recent and fossil turtles. Bulletin of the American Museum of Natural History. 1979;164(2):65–376.

[9] Scheyer TM, Brüllmann B, Sánchez-Villagra MR. The ontogeny of the shell in side-necked turtles, with emphasis on the homologies of costal and neural bones. J Morphol. 2008;269(8):1008–21. doi: 10.1002/jmor.10637 18488992

[10] Lima 10, Santos ALQ, Vieira LG, Da Silva-Junior LM, Romão MF, De Simone SBS, et al. Ontogeny of the shell bones of embryos of Podocnemis unifilis (Troschel, 1848) (Testudines, Podocnemididae). Anatomical Record. 2011;294(4):621–32. doi: 10.1002/ar.21359 21370497

[11] Vieira LG, Santos ALQ, Lima FC, Pinto JGS. Ontogeny of the plastron of the giant Amazon river turtle, Podocnemis expansa (Schweigger, 1812) (Testudines, Podocnemididae). Zoolog Sci. 2009;26(7):491–5. doi: 10.2108/zsj.26.491 19663644

[12] Burke AC, Alberch P. The development and homology of the chelonian carpus and tarsus. J Morphol. 1985;186(1):119–31. doi: 10.1002/jmor.1051860111 30005563

[13] FABREZI M, MANZANO A, ABDALA V, ZAHER H. Developmental basis of limb homology in Pleurodiran turtles, and the identity of the hooked element in the chelonian tarsus. Zoological Journal of the Linnean Society. 2009;155(4):845–66. doi: 10.1111/j.1096-3642.2008.00474.x

[14] Vieira LG, Santos ALQ. Sequence of metacarpal and phalangeal bone formation in embryos of Podocnemis expansa Schweigger, 1812 (Testudines, podocnemididae) stained with alizarin red S. Braz J Morphol Sci. 2007;24(2):104–11.

[15] Vargas-Ramírez M, Caballero S, Morales-Betancourt MA, Lasso CA, Amaya L, Martínez JG. Genomic analyses reveal two species of the matamata (Testudines: Chelidae: Chelus spp.) and clarify their phylogeography. Molecular Phylogenetics and Evolution. 2020;148:106823. doi: 10.1016/j.ympev.2020.106823 32278863

[16] Morales-Betancourt MA, Lasso C, Paez V. Libro rojo de reptiles de Colombia. Bogotá, D. C., Colombia: Instituto de Investigación de Recursos Biológicos Alexander von Humboldt (IAvH), Universidad de Antioquia. 2015.

[17] Lasso CA, Trujillo F, Morales-Betancourt MA, Amaya L, Caballero S, Castañeda B. Conservación y tráfico de la tortuga matamata, Chelus fimbriata (Schneider, 1783) en Colombia: un ejemplo del trabajo conjunto entre el Sistema Nacional Ambiental, ONG y academia. Biota. 2018;19(1):147–59. doi: 10.21068/c2018.v19n01a10

[18] Morales-Betancourt MA, Lasso CA. Chelus fimbriatus. Biología y conservación de las tortugas continentales de Colombia. Instituto de Investigación de Recursos Biológicos Alexander von Humboldt. 2012. 243–6.

[19] Rhodin AGJ, Iverson JB, van Dijk P, Stanford CB, Goode EV, Buhlmann KA, et al. Turtles of the World: Annotated Checklist and Atlas of Taxonomy, Synonymy, Distribution, and Conservation Status. Conservation Biology of Freshwater Turtles and Tortoises: A Compilation Project of the IUCN SSC Tortoise and Freshwater Turtle Specialist Group. Chelonian Research Monographs. 2025. p. 1–575. doi: 10.3854/crm.10.checklist.atlas.v10.2025

[20] Gilbert SF, Loredo GA, Brukman A, Burke AC. Morphogenesis of the turtle shell: the development of a novel structure in tetrapod evolution. Evol Dev. 2001;3(2):47–58. doi: 10.1046/j.1525-142x.2001.003002047.x 11341674

[21] Davis DD, Gore UR, Osgood WH. Clearing and staining skeletons of small vertebrates. Technique series / Field Museum of Natural History. [s.n.]. 1936. doi: 10.5962/bhl.title.5443http://dx.doi.org/10.5962/bhl.title.5443

[22] Vieira LG, Santos ALQ, Moura LR, Orpinelli SRT, Pereira KF, Lima FC. Morphology, development and heterochrony of the carapace of Giant Amazon River Turtle Podocnemis expansa (Testudines, Podocnemidae). Pesq Vet Bras. 2016;36(5):436–46. doi: 10.1590/s0100-736x2016000500014

[23] Wyneken J, Ph D. The anatomy of sea turtles: Introduction. Southeast Fisheries Science Center (U.S.). 2001.

[24] Métrailler S, Gratiet G. Tortues continentales de Guyane française. Switzerland: Bramois. 1996.

[25] Sánchez‐Villagra MR, Winkler JD, Wurst L. Autopodial skeleton evolution in side‐necked turtles (Pleurodira). Acta Zoologica. 2007;88(3):199–209. doi: 10.1111/j.1463-6395.2007.00267.x

[26] Pritchard P. Chelus fimbriata (Schneider 1783) – Matamata Turtle. Conservation Biology of Freshwater Turtles and Tortoises. Chelonian Research Foundation. 2008. p. 020.1-020.10. doi: 10.3854/crm.5.020.fimbriata.v1.2008

[27] Cherepanov G. Morphogenetic and constructional differences of the carapace of aquatic and terrestrial turtles and their evolutionary significance. J Morphol. 2019;280(10):1571–81. doi: 10.1002/jmor.21050 31411770

[28] Lemell P, Lemell C, Snelderwaard P, Gumpenberger M, Wochesländer R, Weisgram J. Feeding patterns of Chelus fimbriatus (Pleurodira: Chelidae). J Exp Biol. 2002;205(Pt 10):1495–506. doi: 10.1242/jeb.205.10.1495 11976360

[29] Cadena E, Link A, Vanegas A, Avellaneda-Otero C, Perdomo C, Urueña-Carrillo D, Sánchez C. Vanegas R. Scheyer T. and Carrillo-Briceño J. New insights into the fossil record of the turtle genus *Chelus* Duméril, 1806 including new specimens with information on cervicals and limb bones. Comptes Rendus Palevol. 2023;22:689–709.doi: 10.5852/CR-PALEVOL2023V22A34

