# Supplementary figures and images for: Gross anatomy of the skeleton of neonates of the Orinoco Matamata turtle (Chelus orinocensis)
Source: PLoS One. 2026 Apr 1;21(4):e0346436. doi: 10.1371/journal.pone.0346436 (PMC13043051; doi:10.1371/journal.pone.0346436)

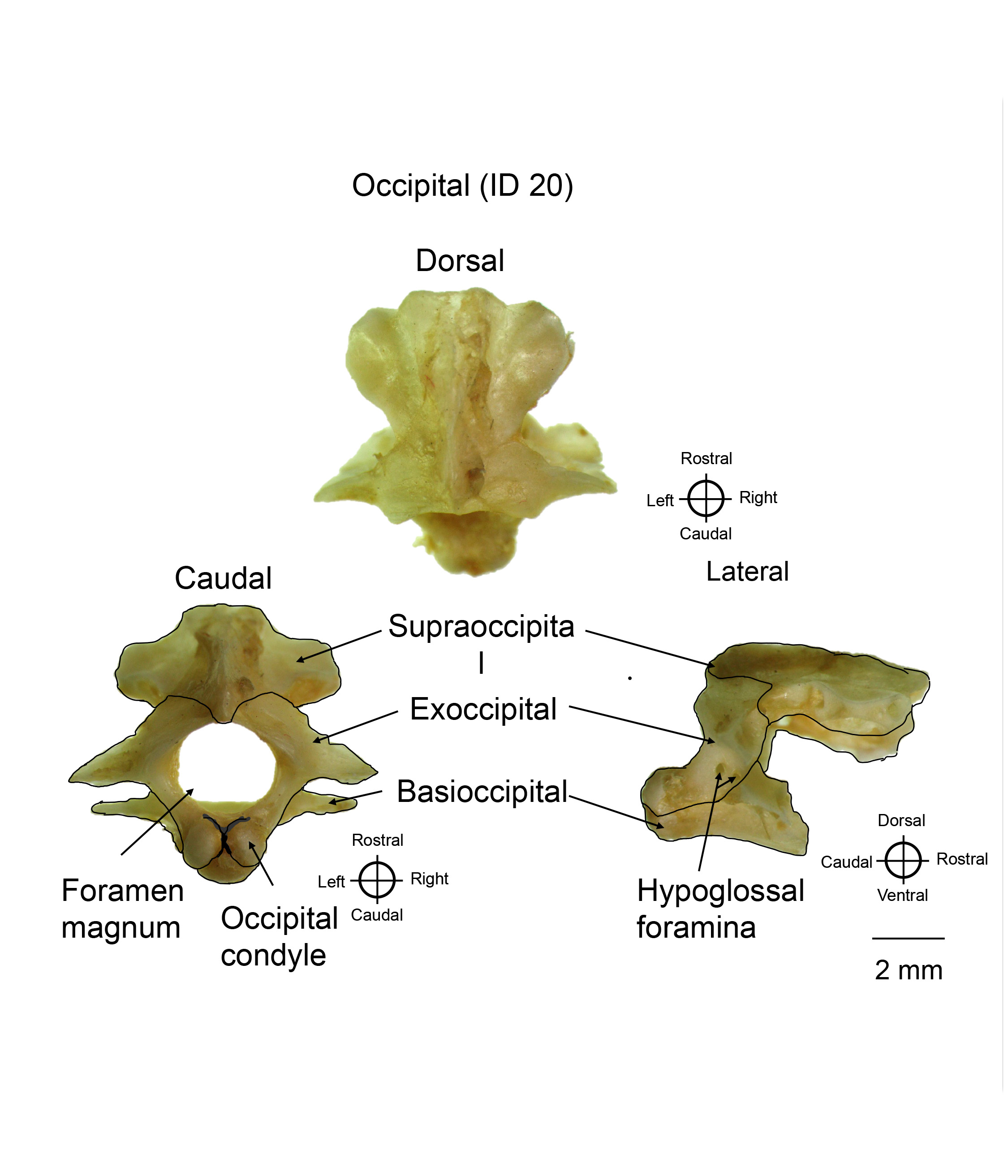

Supplement: S1 Fig — Black lines were added to delineate the bone sutures. (JPG) [file pone.0346436.s001.jpg]

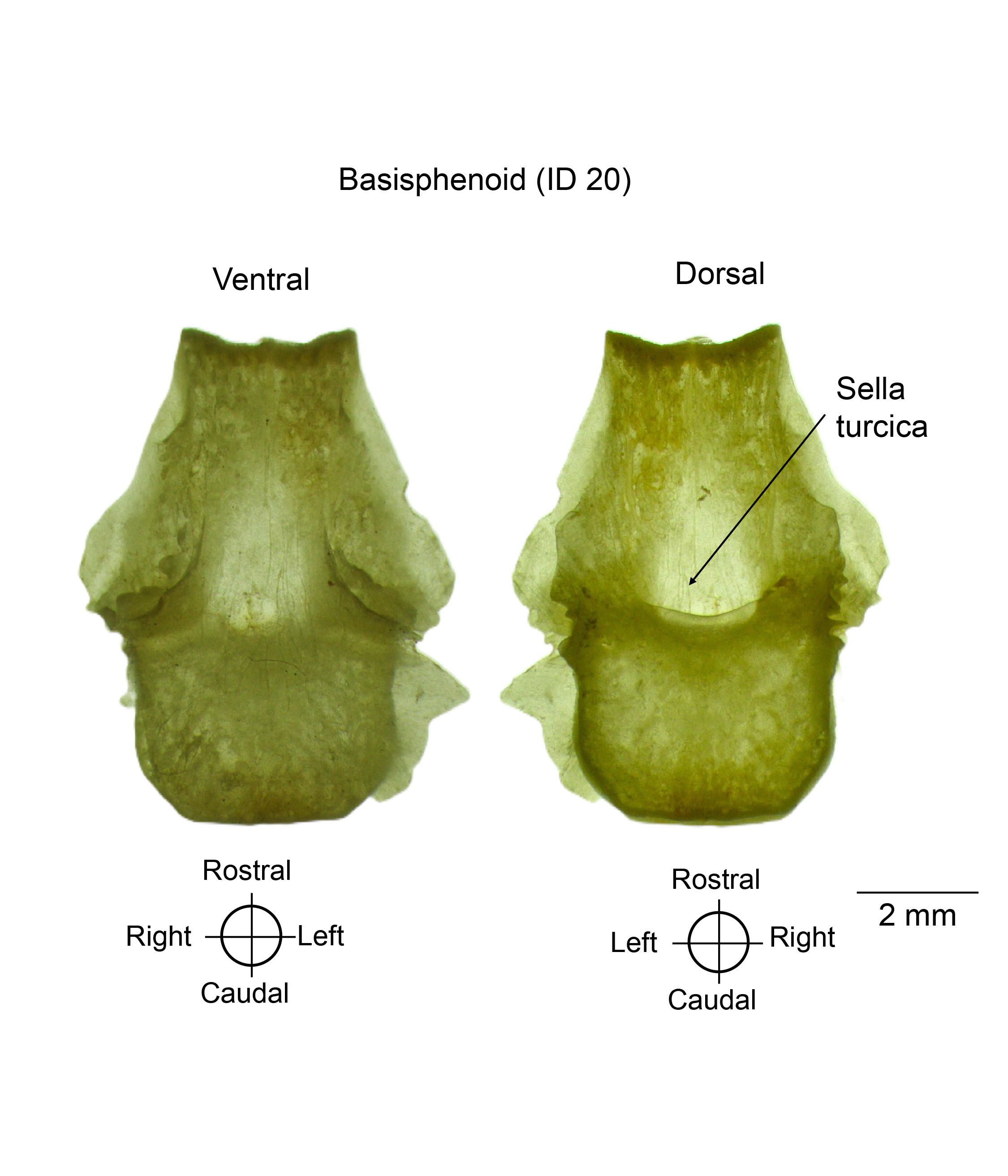

Supplement: S2 Fig — (JPG) [file pone.0346436.s002.jpg]

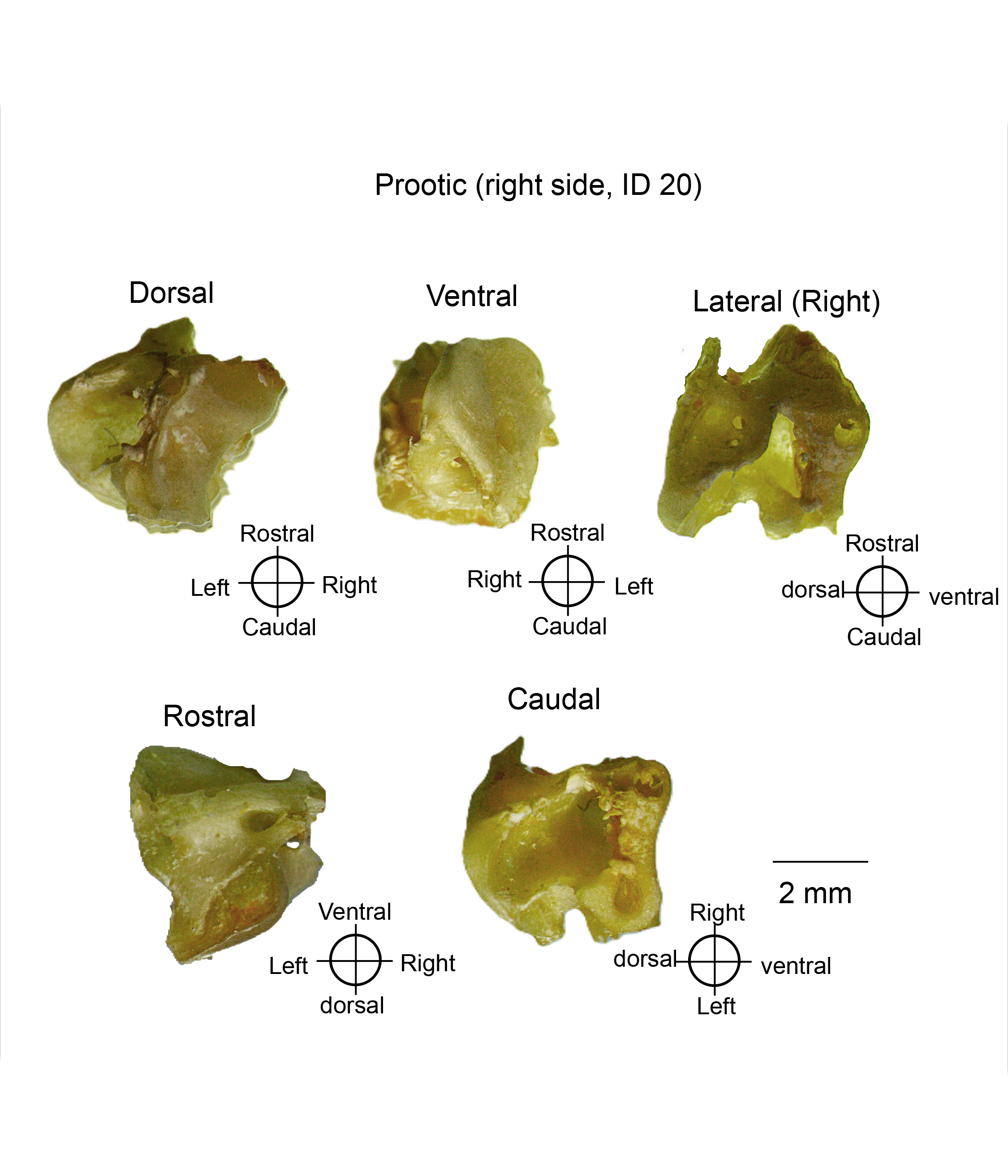

Supplement: S3 Fig — (JPG) [file pone.0346436.s003.jpg]

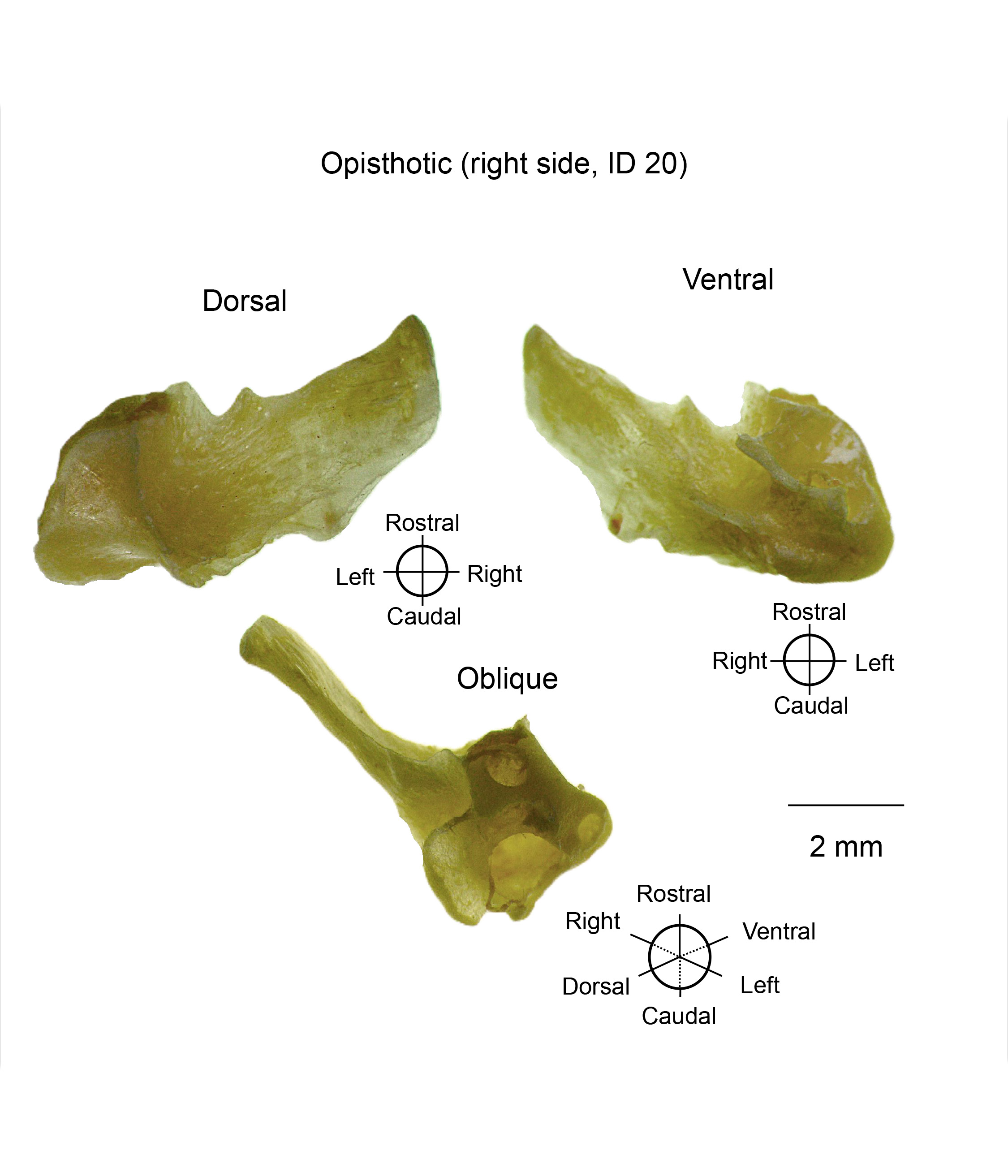

Supplement: S4 Fig — (JPG) [file pone.0346436.s004.jpg]

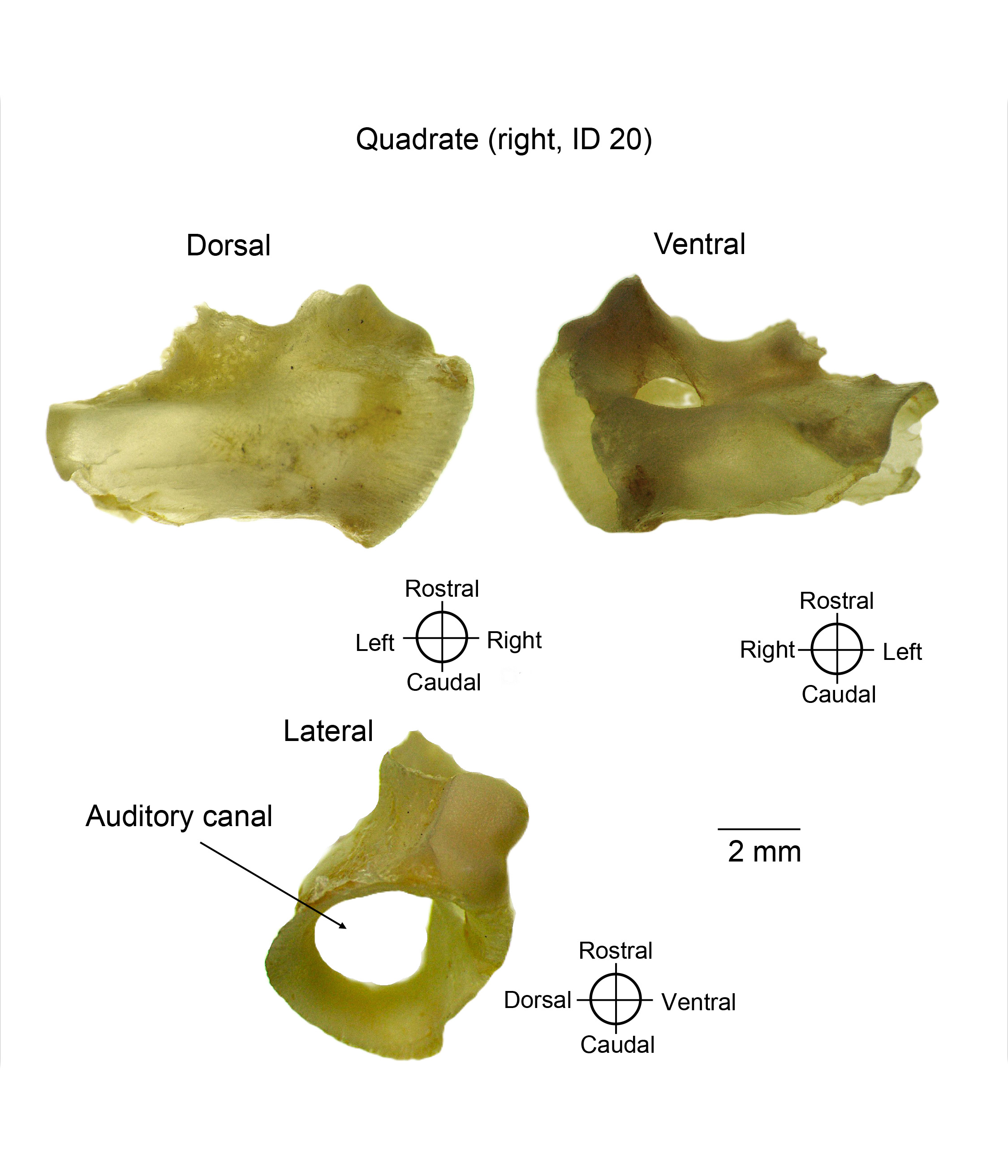

Supplement: S5 Fig — (JPG) [file pone.0346436.s005.jpg]

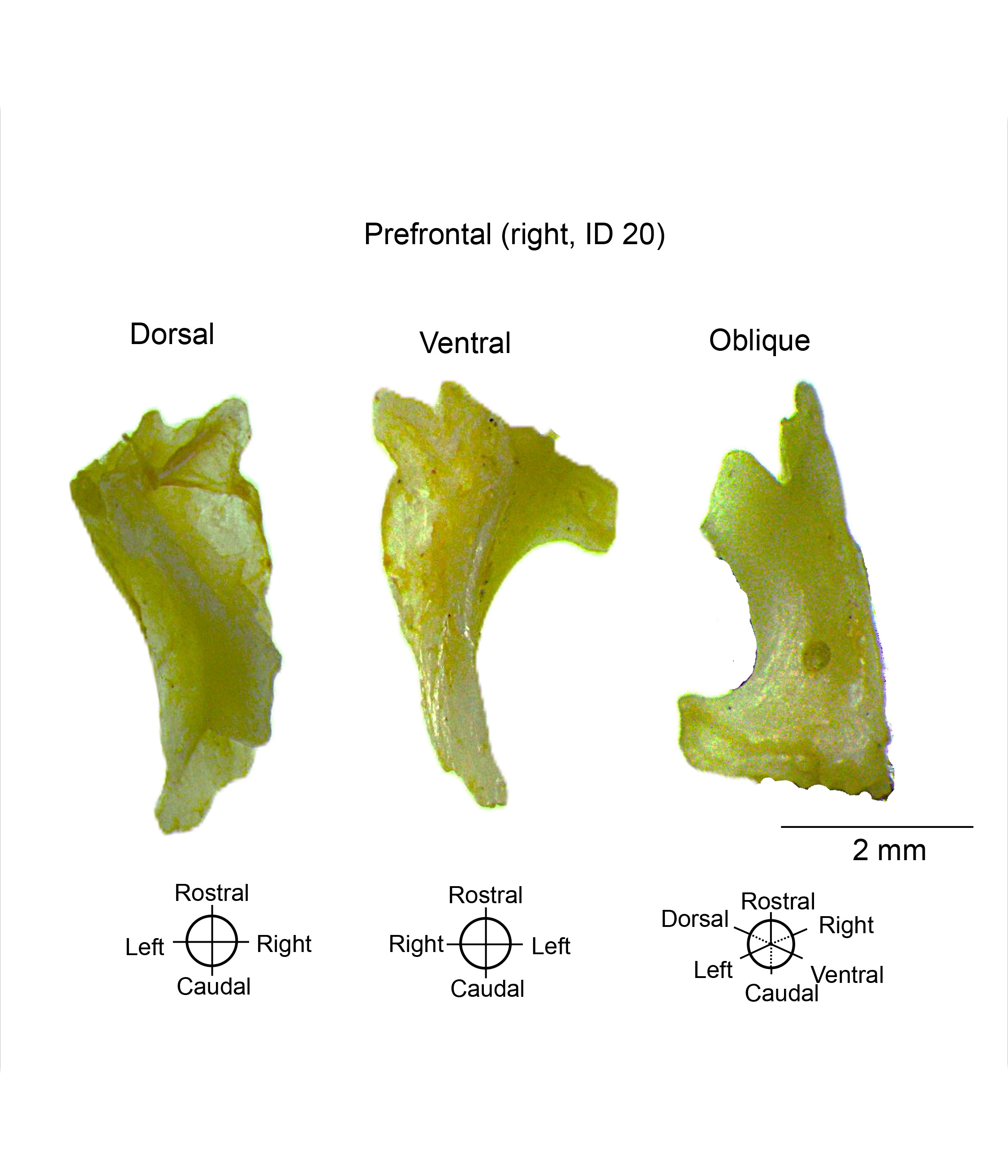

Supplement: S6 Fig — (JPG) [file pone.0346436.s006.jpg]

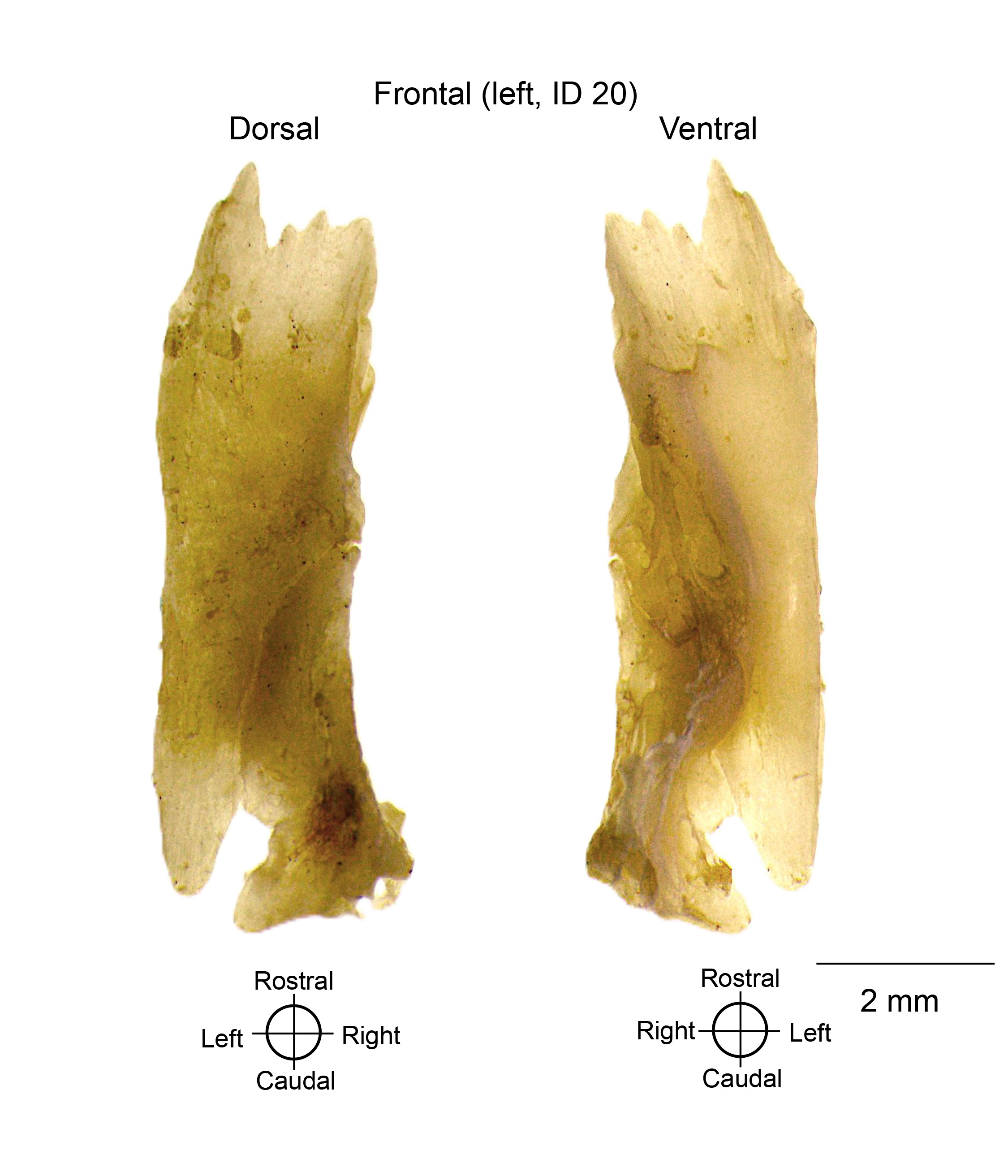

Supplement: S7 Fig — (JPG) [file pone.0346436.s007.jpg]

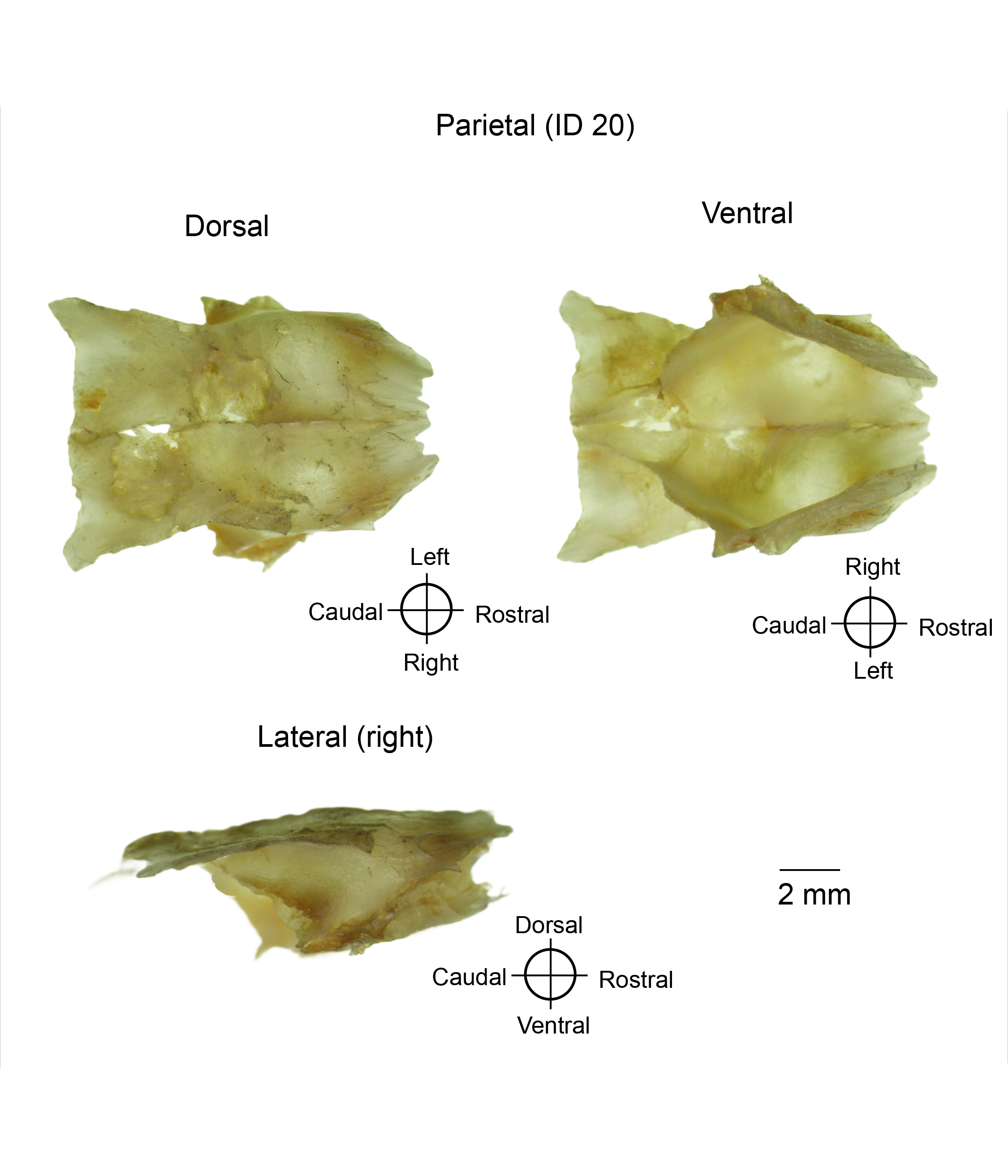

Supplement: S8 Fig — (JPG) [file pone.0346436.s008.jpg]

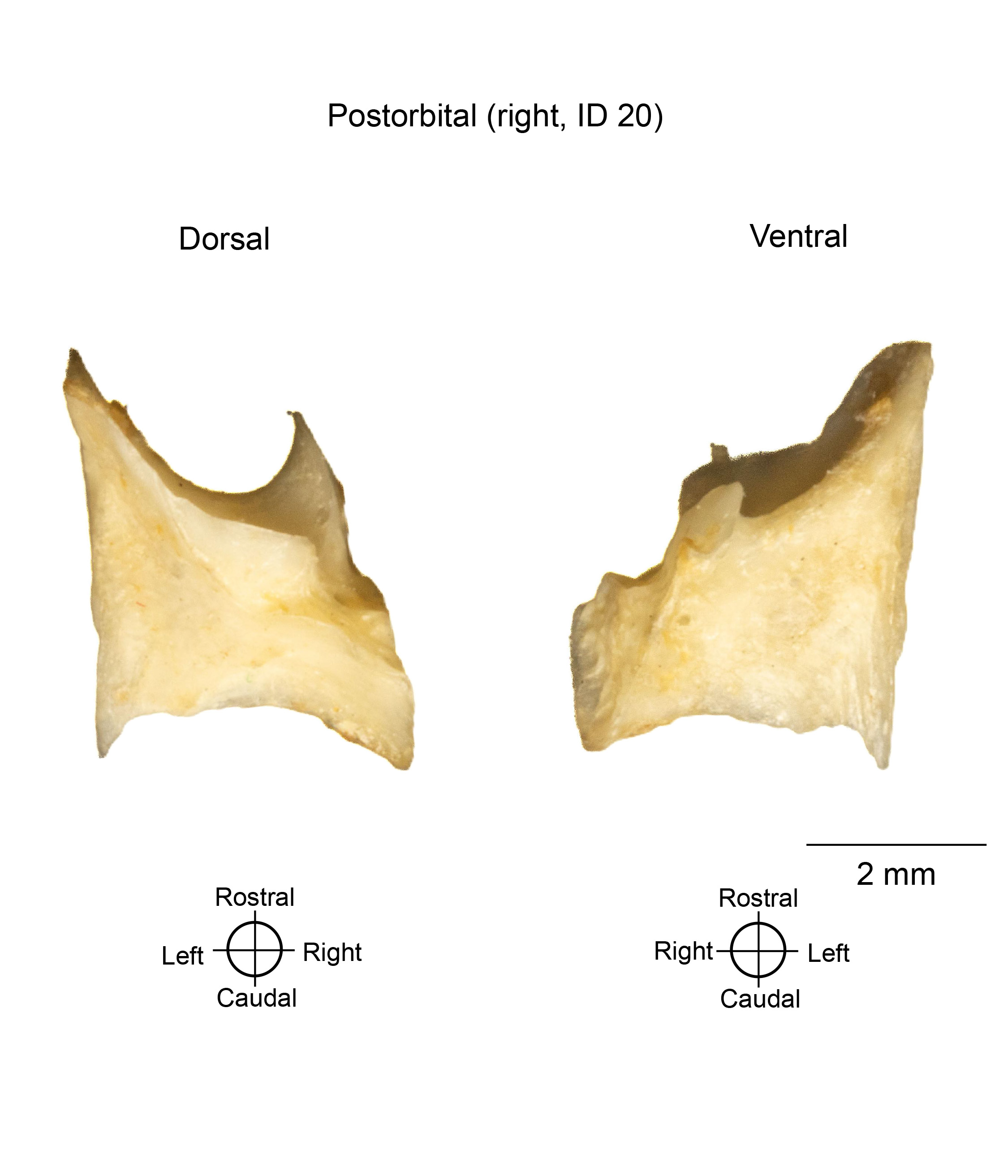

Supplement: S9 Fig — (JPG) [file pone.0346436.s009.jpg]

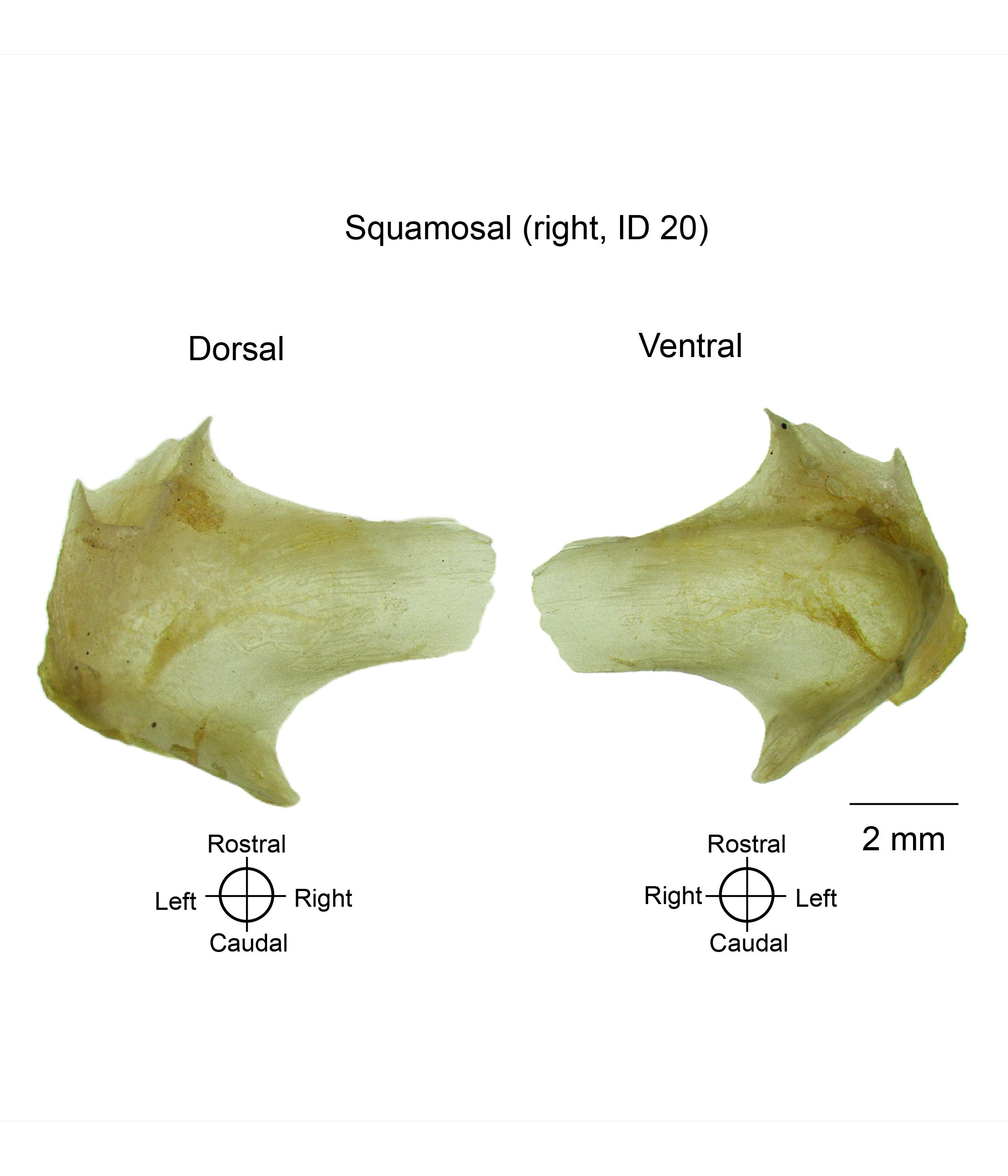

Supplement: S10 Fig — (JPG) [file pone.0346436.s010.jpg]

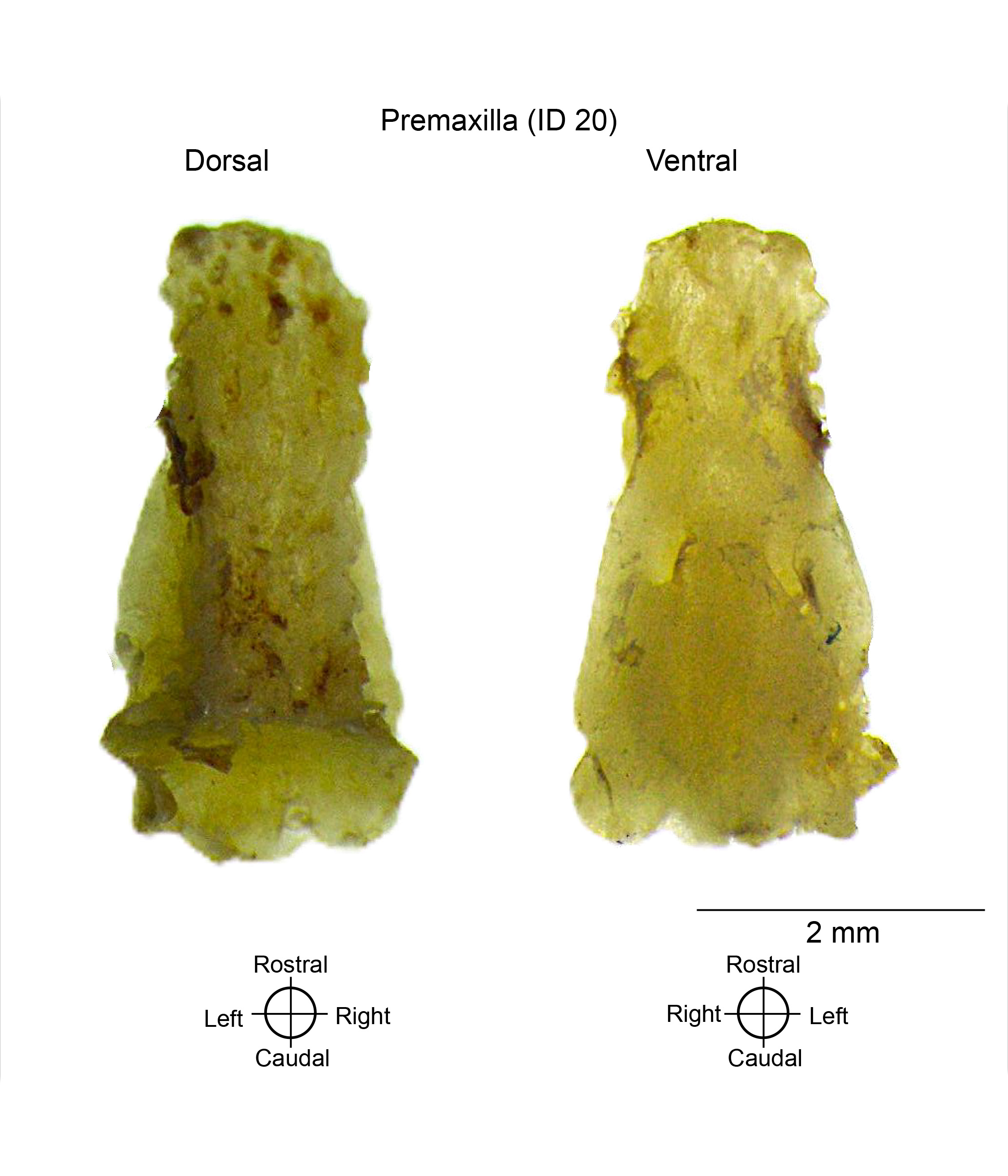

Supplement: S11 Fig — (JPG) [file pone.0346436.s011.jpg]

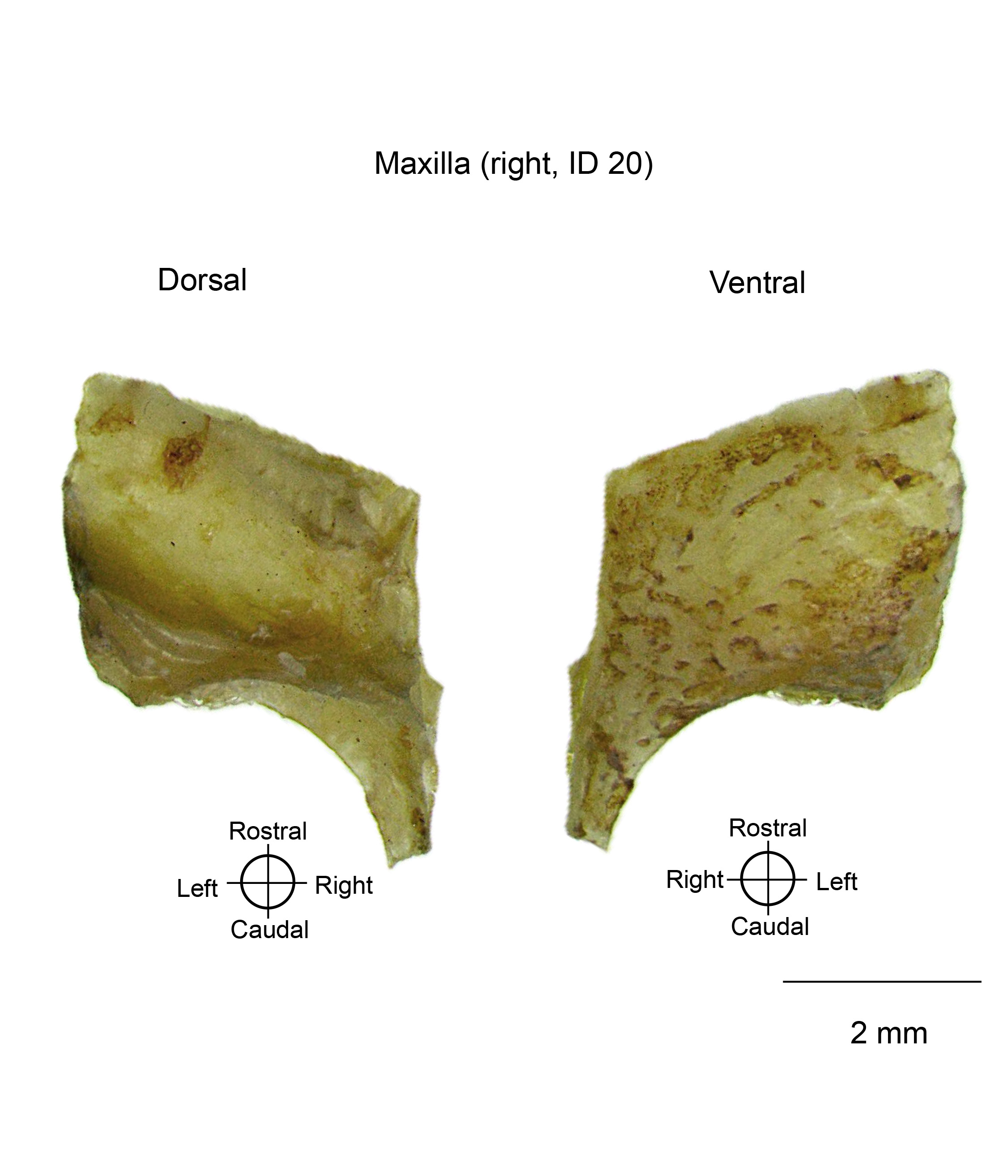

Supplement: S12 Fig — (JPG) [file pone.0346436.s012.jpg]

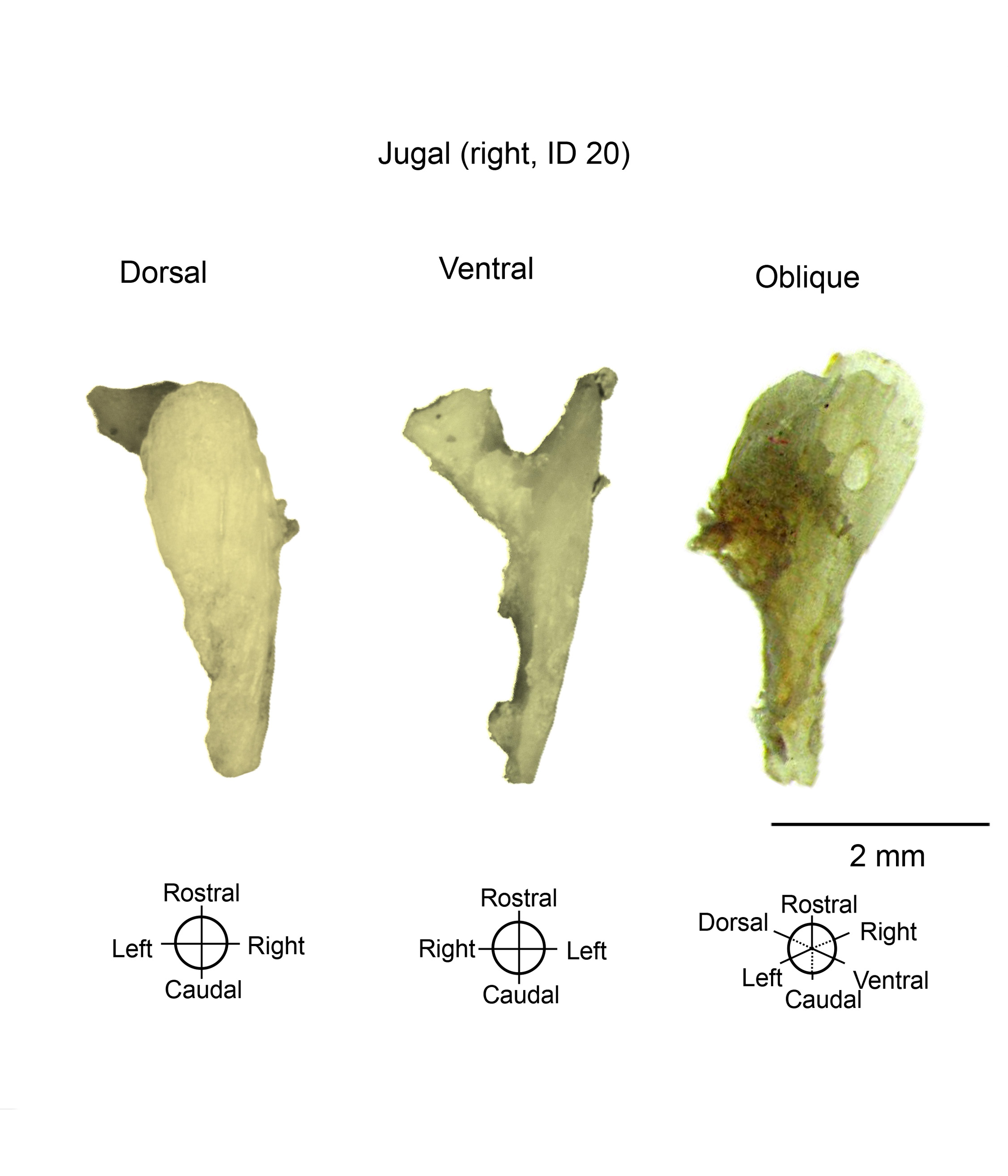

Supplement: S13 Fig — (JPG) [file pone.0346436.s013.jpg]

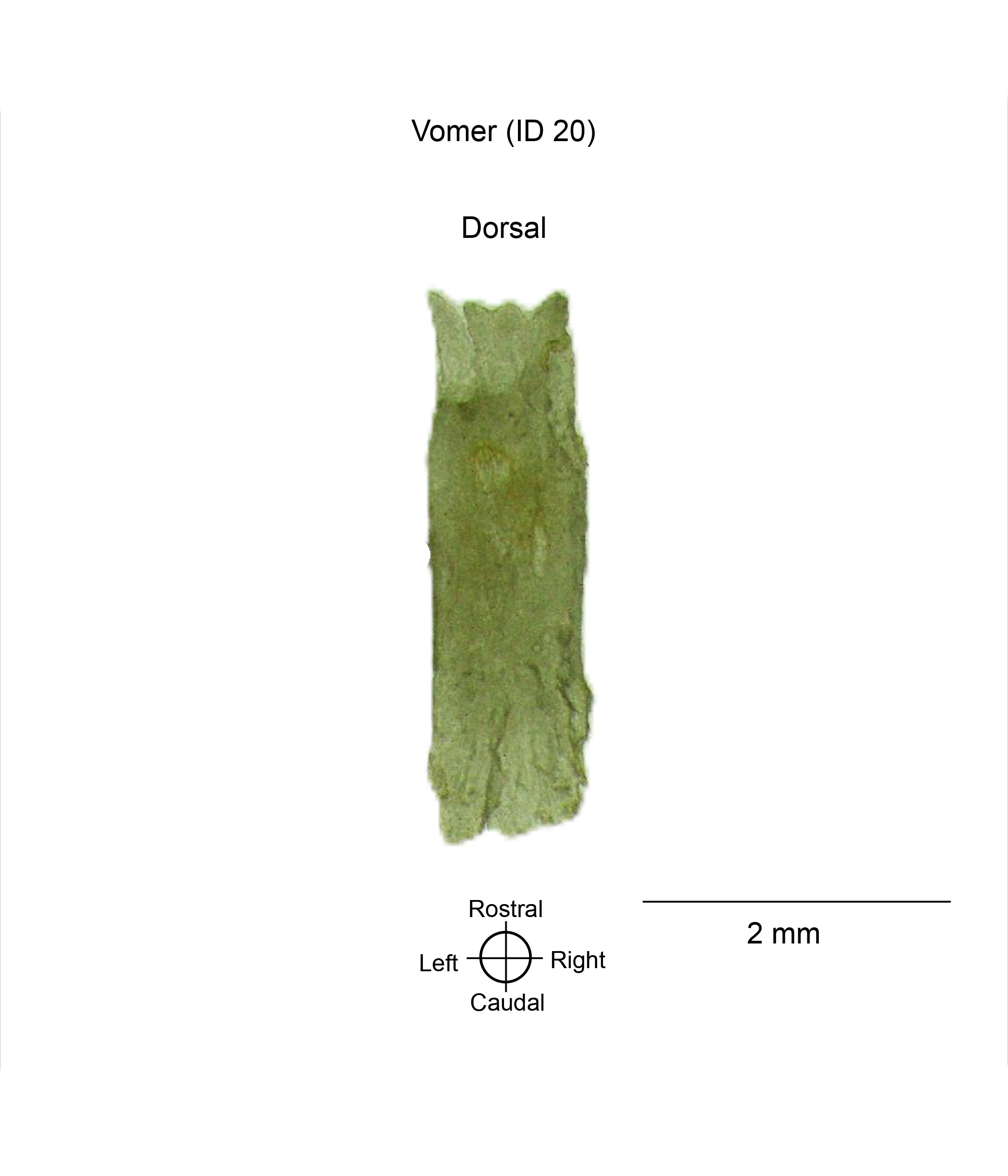

Supplement: S14 Fig — (JPG) [file pone.0346436.s014.jpg]

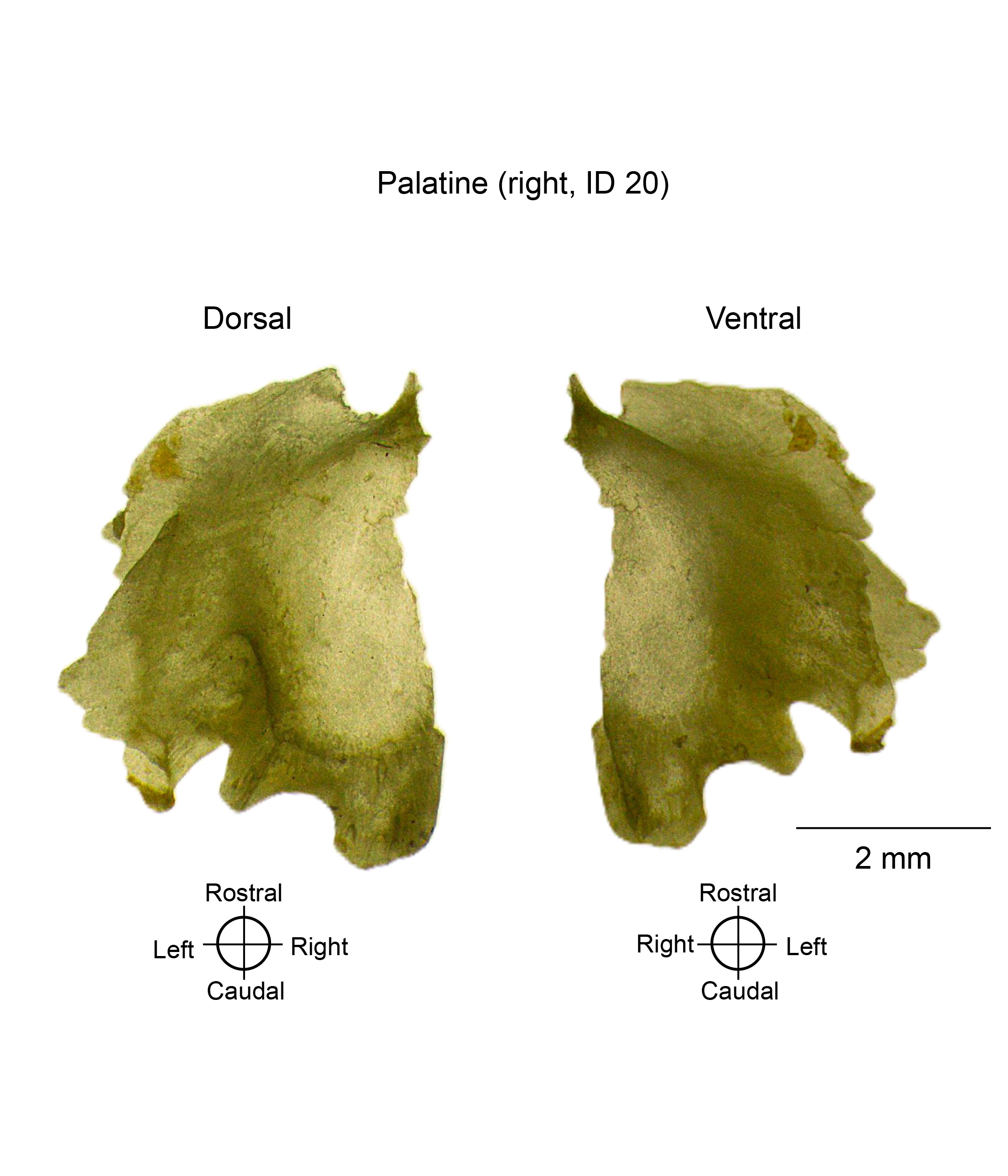

Supplement: S15 Fig — (JPG) [file pone.0346436.s015.jpg]

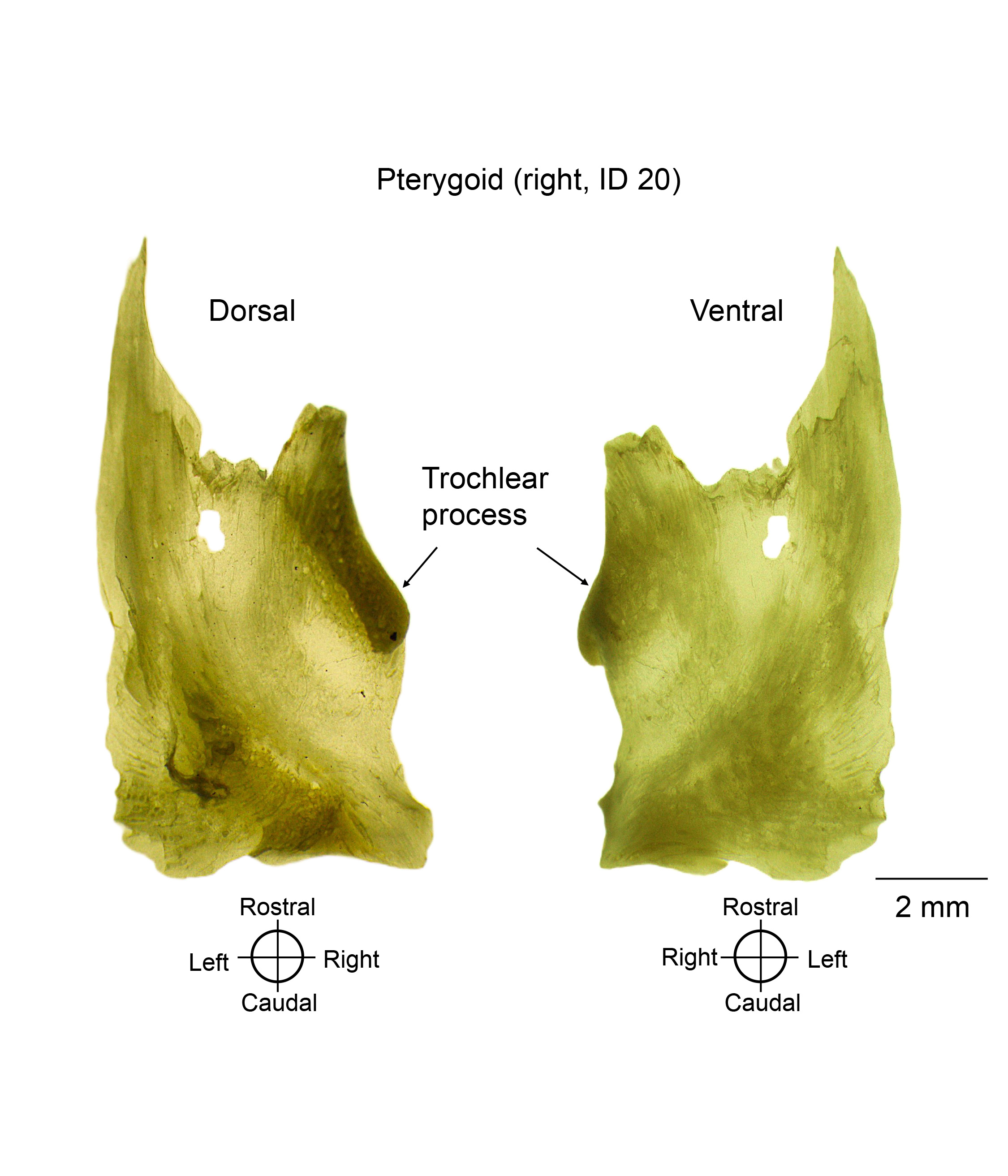

Supplement: S16 Fig — (JPG) [file pone.0346436.s016.jpg]
