# Supplementary material for: Gross anatomy of the skeleton of neonates of the Orinoco Matamata turtle (Chelus orinocensis)
Source: PLoS One. 2026 Apr 1;21(4):e0346436. doi: 10.1371/journal.pone.0346436 (PMC13043051; doi:10.1371/journal.pone.0346436)
Supplement: S1 Table — (DOCX) [file pone.0346436.s017.docx]

| **Regions** | **Bone** | **Paired or unpaired** | **Length (mm)** | **Width (mm)** |
| --- | --- | --- | --- | --- |
| **Neurocranium** | **Skull** |  |  |  |
|  | Basisphenoid | Unpaired | 8.2 | 6.2 |
|  | Basioccipital/exoccipital (occipital) | Unpaired | 4.9 | 8.5 |
|  | Opisthotic | Paired | 7.3 | 7.3 |
|  | Supraoccipital (occipital) | Unpaired | 5.9 | 6.4 |
|  | Prootic | Paired | 3.5 | 3.1 |
| **Splanchnocranium (viscerocranium)** | **Skull** |  |  |  |
|  | Quadrate | Paired | 9.1 | 6.3 |
|  | **Mandible** |  |  |  |
|  | Articular | Paired | 2.8 | 2.9 |
|  | **Hyoid apparatus** |  |  |  |
|  | Corpus hyoidis | Unpaired | 10.7 | 4.8 |
|  | Cornu branchiale I | Paired | 13.0 | 6.0 |
|  | Cornu branchiale II | Paired | 17.4 | 2.0 |
|  | Hypoglossum | Unpaired | 11.3 | 9.6 |
| **Dermatocranium** | **Skull** |  |  |  |
|  | Prefrontal | Paired | 3.7 | 2.3 |
|  | Frontal | Paired | 8.9 | 3.9 |
|  | Parietal | Unpaired | 12.6 | 4.0 |
|  | Premaxilla | Unpaired | 4.4 | 2.3 |
|  | Maxilla | Paired | 5.8 | 2.7 |
|  | Jugal | Paired | 2.4 | 2.6 |
|  | Squamosal | Paired | 7.7 | 6.2 |
|  | Postorbital | Paired | 6.5 | 3.9 |
|  | Palatine | Paired | 5.1 | 3.8 |
|  | Vomer | Unpaired | 3.7 | 1.0 |
|  | Pterygoid | Paired | 12.5 | 6.4 |
|  | **Mandible** |  |  |  |
|  | Angular | Paired | 5.2 | N/A |
|  | Coronoid | Paired | 2.9 | 2.0 |
|  | Dentary | Paired | 9.4 | 5.4 |
|  | Splenial | Paired | 4.5 | N/A |
|  | Surangular | Paired | 6.5 | 2.0 |
|  | **Hyoid apparatus** |  |  |  |
|  | Entoglossal | Paired | 5.4 | 2.4 |
| **Axial skeleton** | **Cervical vertebrae** |  |  |  |
|  | C1 | Unpaired | 4.6 | 3.7 |
|  | C2 | Unpaired | 8.3 | 4.7 |
|  | C3 | Unpaired | 7.3 | 5.6 |
|  | C4 | Unpaired | 6.1 | 5.0 |
|  | C5 | Unpaired | 6.2 | 4.6 |
|  | C6 | Unpaired | 6.3 | 5.0 |
|  | C7 | Unpaired | 10.5 | 5.5 |
|  | C8 | Unpaired | 9.2 | 6.3 |
|  | **Dorsal vertebrae** |  |  |  |
|  | D1 - D10 | Unpaired | NA |  |
|  | **Sacral verebrae** |  |  |  |
|  | S1 - S2 | Unpaired | NA |  |
|  | **Caudal vertebrae** |  |  |  |
|  | Ca1 - Ca16-18 | Unpaired | NA |  |
|  | **Dermal bones of carapace** |  |  |  |
|  | Nuchal | Unpaired | NA |  |
|  | Neural bones (7) | Unpaired | NA |  |
|  | Pleural bones (8) | Paired | NA |  |
|  | Suprapygal | Unpaired | NA |  |
|  | Pygal | Unpaired | NA |  |
|  | Peripheral bones (11) | Paired | NA |  |
|  | **Ribs** 1-10 | Paired | NA |  |
|  | **Plastron** |  |  |  |
|  | Epiplastra | Paired | 17.4 | 6.1 |
|  | Hyoplastra | Paired | 24.8 | 10.1 |
|  | Hypoplastra | Paired | 23.9 | 6.6 |
|  | Xiphiplastra | Paired | 18.3 | 5.2 |
|  | Entoplastron | Unpaired | 13.8 | 7.8 |
| **Appendicular skeleton** | **Pectoral girdle** |  |  |  |
|  | Scapula | Paired | 14.5 | 1.3 |
|  | Acromial process | Paired | 9.6 | 1.6 |
|  | Coracoid | Paired | 9.1 | 5.8 |
|  | **Forelimb** |  |  |  |
|  | Humerus | Paired | 12.6 | 1.7 |
|  | Radius | Paired | 6.9 | 0.7 |
|  | Ulna | Paired | 6.6 | 1.4 |
|  | Carpals (1-8) | Paired | NA |  |
|  | Metacarpal I | Paired | 2.8 | 2.4 |
|  | Metacarpal II | Paired | 3.4 | 3.8 |
|  | Metacarpal III | Paired | 4.4 | 4 |
|  | Metacarpal IV | Paired | 3.2 | 2.8 |
|  | Metacarpal V | Paired | 2.4 | 2.4 |
|  | Phalanges (2-3-3-3-3) | Paired | NA |  |
|  | **Pelvic girdle** |  |  |  |
|  | Ilium | Paired | 6.3 | 2.4 |
|  | Ischium | Paired | 5.3 | 2.2 |
|  | Pubis | Paired | 4.8 | 3 |
|  | **Hindlimb** |  |  |  |
|  | Femur | Paired | 14.3 | 1.7 |
|  | Tibia | Paired | 10.2 | 1.3 |
|  | Fibula | Paired | 10 | 0.9 |
|  | Tarsals (5) | Paired | NA |  |
|  | Metatarsal I | Paired | 3.5 | 3 |
|  | Metatarsal II | Paired | 5.2 | 4.9 |
|  | Metatarsal III | Paired | 5.4 | 5.7 |
|  | Metatarsal IV | Paired | 5.1 | 5.3 |
|  | Metatarsal V | Paired | 4.1 | 4 |
|  | Phalanges (2-3-3-3-3) | Paired | NA |  |
